# Therapeutic NAMPT inhibition reveals a targetable metabolic vulnerability in neuroblastoma

**DOI:** 10.1186/s13046-026-03793-5

**Published:** 2026-08-05

**Authors:** Sophia Varriano, Amy Yu, Abantika Chakraborty, Ariana E. Nelson, Amy James, Kristine Isanogle, Nimit Patel, Caleb Kim, Unsun Lee, Victor J. Collins, Grace B. McKay-Corkum, Ye Yang, Xiaohu Zhang, Crystal McKnight, Kelli Wilson, Carleen Klumpp-Thomas, Michele Ceribelli, David Holland, Ming Sun, Gitanjali Asampille, Ying Wu, Krithika Bhuvaneshwar, Brad Gouker, Donna Butcher, Bhushan L. Thakur, Arnulfo Mendoza, Sameer H. Issaq, Mirit I. Aladjem, Baktiar O. Karim, Jack F. Shern, Parthav Jailwala, Simone Difilippantonio, Craig J. Thomas, Daniel R. Crooks, Rosa Nguyen, Carol J. Thiele, Christine M. Heske

**Affiliations:** 1 Pediatric Oncology Branch, Center for Cancer Research, National Cancer Institute, National Institutes of Health, 10 Center Drive, CRC, Room 1W-3816, Bethesda, MD 20892 USA; 2 Animal Research Technical Support, Laboratory Animal Sciences Program, Leidos Biomedical Research, Inc., Frederick National Laboratory for Cancer Research, Frederick, MD USA; 3 Small Animal Imaging Program, Laboratory Animal Sciences Program, Leidos Biomedical Research, Inc., Frederick National Laboratory for Cancer Research, Frederick, MD USA; 4 Clinical Cancer Metabolism Facility, Urologic Oncology Branch, Center for Cancer Research, National Cancer Institute, National Institutes of Health, Bethesda, MD USA; 5 Division of Preclinical Innovation, National Center for Advancing Translational Sciences, National Institutes of Health, Rockville, MD USA; 6 Advanced Biomedical Computational Science, Frederick National Laboratory for Cancer Research, Frederick, MD USA; 7 Molecular Histopathology Laboratory, Laboratory Animal Sciences Program, Frederick National Laboratory for Cancer Research, Frederick, MD USA; 8 Developmental Therapeutics Branch, Center for Cancer Research, National Cancer Institute, National Institutes of Health, Bethesda, MD USA; 9 Urologic Oncology Branch, Center for Cancer Research, National Cancer Institute, National Institutes of Health, Bethesda, MD USA

**Keywords:** Neuroblastoma, NAMPT, NAMPT inhibition, NAD^+^ salvage, Metabolic vulnerability, DNA damage, Orthotopic xenograft

## Abstract

**Background:**

Neuroblastoma (NB) remains a major cause of pediatric cancer mortality for which new therapeutic strategies are needed. Exploitation of reprogrammed metabolic pathways offers an opportunity for cell-type specific anticancer therapeutics. To identify pediatric solid tumors with an enhanced susceptibility to targeting the nicotinamide adenine dinucleotide (NAD^+^) salvage pathway, we performed an unbiased high-throughput screen of over 200 cancer cell line models using inhibitors of nicotinamide phosphoribosyltransferase (NAMPT), the rate limiting enzyme in the NAD^+^ salvage pathway. Our analysis identified NB as one of the most sensitive solid tumor types, prompting further investigation of NAMPT inhibition as a potential therapeutic strategy in this disease.

**Methods:**

Using two early phase clinical NAMPT inhibitors (OT-82 and KPT-9274), we validated screen results using assays of proliferation and survival in a panel of molecularly diverse NB cell lines. Effects on proliferation, survival, NAD^+^ abundance, adenosine triphosphate (ATP) levels, and energy-related metabolites were quantified, and downstream consequences of NAD^+^-consuming enzymatic pathways were examined. Tolerability, antitumor activity and pharmacodynamic effects of OT-82 were evaluated in three orthotopic NB mouse models.

**Results:**

In the drug screen, NB models ranked among the most sensitive pediatric solid tumor cells lines to NAMPT inhibition. OT-82 and KPT-9274 potently suppressed proliferation and viability across multiple molecularly diverse NB models in an on-target manner. Mechanistically, NAMPT inhibition resulted in rapid depletion of NAD^+^ and ATP, disruption of energy metabolism, accumulation of DNA damage, and induction of irreversible non-apoptotic cellular death. In vivo, OT-82 was well tolerated and produced marked antitumor activity, including tumor regressions in orthotopic NB models, including several with regional liver metastases. Pharmacodynamic analysis confirmed intratumoral NAD^+^ depletion during treatment, demonstrating on-target pathway inhibition in vivo.

**Conclusions:**

These findings identify NB as a highly NAMPT inhibitor-sensitive pediatric solid tumor and establish NAMPT-dependent NAD^+^ biosynthesis as a targetable metabolic vulnerability in this disease. By integrating an unbiased discovery screen with mechanistic and orthotopic in vivo validation using clinically relevant inhibitors, this study provides a strong translational rationale for clinical investigation of NAMPT inhibitors in NB.

**Supplementary Information:**

The online version contains supplementary material available at https://doi.org/10.1186/s13046-026-03793-5.

## Background

Metabolic reprogramming is a hallmark of cancer due to increased demands of cellular growth and proliferation in malignant cells. Cancer cells display cell-type specific dependencies on particular metabolic pathways to sustain rapid growth, and these dependencies represent potential therapeutic opportunities [1–4]. One such opportunity is targeting the production of nicotinamide adenine dinucleotide (NAD^+^), an essential cofactor for enzymes in energy metabolism and other downstream processes [5–7]. Three redundant pathways produce NAD^+^,Additional methods for supplemental dataeach using different enzymes and precursors to generate NAD^+^. The de novo pathway converts tryptophan via quinolinate phosphoribosyltransferase (QPRT), the Preiss-Handler pathway synthesizes NAD^+^ from nicotinic acid (NA) via nicotinate phosphoribosyltransferase (NAPRT), and the salvage pathway converts nicotinamide (NAM) to nicotinamide mononucleotide (NMN) via nicotinamide phosphoribosyltransferase (NAMPT) [7, 8]. Although generally redundant in normal cells, these pathways may be altered in cancer cells, resulting in differential dependencies on each pathway [9]. In addition, cancer cell types with increased NAD^+^ demands may be more dependent on the salvage pathway [7, 10, 11], making targeting NAMPT, the only clinically targetable NAD^+^ production enzyme, a potential therapeutic opportunity. While several NAMPT inhibitors (NAMPTis) have been tested in early phase clinical trials in adult cancers [12–18], they have yet to be tested in pediatric cancers.

Our group and others have previously reported that NAMPTis are highly effective against models of two pediatric sarcomas, Ewing sarcoma (EwS) and rhabdomyosarcoma (RMS) [19–23]. Compared to multiple other types of adult solid tumors, these pediatric models were more sensitive to NAMPTis with IC_50_ values more than 10- to 20-fold lower, suggesting that antitumor activity of NAMPTis may occur at lower doses in these pediatric sarcomas than the doses needed to affect other solid tumors [19, 23]. As pediatric cancers represent a group of cancers of high unmet clinical need, we sought to investigate if other pediatric solid tumors exhibit similarly high sensitivity to NAMPTis. Using an unbiased high-throughput cancer cell line screen of over 200 cell lines, we identified that neuroblastoma (NB) is one of the most NAMPTi-sensitive solid tumor types, with sensitivity comparable or greater than that observed in pediatric sarcoma models. This finding prioritized NB for further translational investigation. NB is the most common extracranial solid tumor in children, accounting for 15% of all pediatric cancer deaths [24]. Despite intense multimodal therapy, the prognosis for patients with high-risk NB is poor with an overall survival rate of less than 50% [25–27]. Based on the results of our drug screen, we sought to validate this finding across a broad panel of in vitro and in vivo NB models and to define the mechanistic effects of therapeutic NAMPT inhibition in NB, given that downstream effects of NAD^+^ depletion are cell-type specific [28, 29]. We further sought to determine whether this screen-identified vulnerability could be supported by pharmacodynamic and efficacy studies in in vivo orthotopic NB models.

Herein, we demonstrate a potent class effect of NAMPTis as effective agents against NB models. In vitro, treatment with the NAMPTi OT-82 in NB cells resulted in depletion of NAD^+^ and adenosine triphosphate (ATP), arrest of NAD^+^-dependent metabolic pathways, as well as other NAD^+^ dependent downstream processes, resulting in accumulation of DNA damage and non-apoptotic cell death. In vivo*,* we observed regression of multiple orthotopic xenograft models upon OT-82 treatment as well as a reduction in intratumoral NAD^+^ levels. Together, these findings establish NB as a clinically actionable indication for NAMPT inhibition and provide strong preclinical rationale for clinical study of NAMPTis in this disease.

## Materials and methods

Key reagents, including cell lines, compounds, antibodies, and commercial assays are detailed in Supplemental Table S1. Additional methods for supplemental data can be found in Supplementary Materials and Methods.

### Cell line screening dataset

Quantitative high-throughput screening was completed using previously reported methods [30–32]. Briefly, cell models including BE(2)C, SY5Y, and Kelly cells were seeded in 5 µL of growth media using a Multidrop Combi dispenser into 1536-well white polystyrene tissue culture-treated Corning plates at standard cell densities (500–1000 cells/well) for analysis during exponential growth. Following addition of cells, 23 nL of Mechanism Interrogation Plate (MIPE) 6.0 compounds were added to individual wells (11 doses tested per compound) via a 1536 pin-tool. Plates were incubated for 48 h at standard incubator conditions covered by a stainless steel gasketed lid to prevent evaporation. 3 μL of Cell Titer-Glo (Promega) was added to each well; plates were incubated at room temperature for 15 min with stainless-steel lid. Luminescence readings were taken using a Viewlux (PerkinElmer) with a 2 s exposure time per plate. Compound dose–response curves were normalized to DMSO and empty well controls on each plate.

### Cell lines

Details about NB cell lines (SK-N-AS, BE(2)C, Kelly, SY5Y, IMR-32, IMR-5, KCNR, and LAN5 are in Supplemental Table S1 and have been previously reported [33, 34]. Patient-derived xenograft (PDX)-derived cell lines SJNBL012407 and SJNBL013762 were derived from PDX models obtained from St. Jude Children’s Solid Tumor Network and have been previously described [35]. Cell lines were confirmed to be mycoplasma negative by PCR and authenticated by short tandem repeat DNA fingerprinting prior to experimental use. Cells were maintained in RPMI growth medium with 10% FBS, heat-inactivated, penicillin, streptomycin, and 1% L-glutamine at 37 °C in an atmosphere of 5% CO_2_.

### Compounds

Details about NAMPTis (OT-82, FK-866, KPT-9274, and GNE-618), and NMN are in Supplemental Table S1. Stock solutions for in vitro experiments were prepared in DMSO, aliquoted, and stored at −20 °C. For in vivo studies, OT-82 was prepared weekly by dissolving in a solution of 30% (2)-Hydroxypropyl-B-cyclodextrin (HPBCD) (MilliporeSigma) with 40 mM HCl.

### Immunoblotting

For necroptosis evaluation, all cells were plated at 1 × 10^6^/10-cm overnight before treatment with OT-82 for 48 h. For autophagy studies, SK-N-AS and BE(2)C were plated at 1 × 10^4^/6-well plate; Kelly and SY5Y were seeded at 2 × 10^4^/6-well plate before treatment with OT-82 (24, 48, or 72 h). For all other experiments, SK-N-AS and BE(2)C cells were plated at 1 × 10^6^/10-cm plate; Kelly, SY5Y, IMR5, IMR32, LAN5, KCNR, SJNBL012407, and SJNBL013762 cells were plated at 2 × 10^6^/10-cm plate. Upon harvest, cells were washed with ice-cold PBS and lysed with RIPA cell lysis buffer with phosphatase/protease inhibitors. For necroptosis and autophagy studies, lysates were sonicated at 20 Hz for 5 s and quantified using Nanodrop Spectrophotometer at 280 nm. Protein lysates (30 μg/lane) were separated in 4%−12% SDS-PAGE and transferred to nitrocellulose membranes (ThermoFisher). Membranes were blocked with 5% nonfat dry milk in TBS (KPL)-Tween 20 (MilliporeSigma; 25 mm Tris–HCl, pH 7.4; 0.13 M of sodium chloride; 0.1% Tween 20) or 5% BSA (necroptosis evaluations only). Blots were incubated with primary antibodies (Supplemental Table S1). Bands were visualized on a BioRad Image Lab camera using Pico ECL detection reagent (ThermoFisher).

### Cell proliferation assays

IncuCyte live-cell analysis systems (Sartorius) were used to assess real-time proliferation of NB cells. NB cells were plated at 5,000–13,000 cells/well in 96-well plates overnight and treated the following day.

### NAD^+^/NADH-Glo assay

Cells were plated at 1–2 × 10^6^/10-cm plate and treated the following day. For NMN rescue, NMN and OT-82 were co-administered. Cells were harvested at respective timepoints as previously described [23]. NAD^+^ and NADH were quantified using the NAD^+^/NADH-Glo Assay (Promega) per manufacturer’s instructions.

### Intracellular metabolite analyses

Targeted analysis of intracellular metabolites was performed using cells cultured on 6-well plates in 2 mL of culture medium/well. Additional wells with culture medium only were incubated to generate procedural blank samples. Following 24 h treatment with OT-82 or DMSO, media was aspirated and wells were rapidly washed with 5 mL ice cold ddH_2_O, after which 200 μL of −80 °C UHPLC-grade methanol (Sigma Aldrich) was added to each well and plates were incubated at −20 °C for 5 min. Subsequently 200 μL of ddH_2_O was added to the wells, wells were scraped with cell lifters, and extracts collected in pre-labeled Eppendorf tubes. Samples were centrifuged at 20 K x g for 10 min at 4 °C, and 270 μL of the supernatant was applied to doubly pre-washed 10 K MWCO centrifugal filters (Amicon) and centrifuged at 14 K x g for 30 min. The resulting eluate was immediately frozen in liquid nitrogen and lyophilized overnight. Cell pellet was evaporated to dryness in a SpeedVac and analyzed for total cellular protein content using bicinchoninic acid (BCA) method. For 1D^1^H NMR analysis, polar metabolite residues were dissolved in NMR buffer and analyzed as previously described [36]. Ultrahigh resolution ion chromatography-mass spectrometry was performed as previously described [37].

### CellTiter-Glo 2.0 assay

Cells were prepared as described for the NAD^+^/NADH-Glo Assay. ATP was measured using the CellTiter-Glo 2.0 Assay (Promega) per manufacturer’s instructions.

### Extracellular flux analysis

Seahorse XFe96 Extracellular Flux Analyzer (Agilent) was used to analyze cellular bioenergetics. Cells were plated in XF 96-well plates (Agilent) at 5,000 (BE(2)C or 10,000 cells/well (SK-N-AS, SY5Y, and Kelly) at 37 °C, 5% CO2 for 6 h before being treated with DMSO or OT-82 for 22 h. Assay media was prepared according to manufacturer’s instructions, however OT-82 was added to assay media for wells containing treated cells. Measurements of basal extracellular acidification rate (ECAR) and oxygen consumption (OCR) rate were performed per manufacturer’s instructions.

### Sirtuin activity assay

Cells were plated at 1–2 × 10^6^/10-cm plate and adhered overnight before treatment and then harvesting at 24- and 48 h post treatment using 0.25% trypsin (ThermoFisher). Total cell extracts were prepared in NP40 buffer. SIRT-1 specific activity was measured using the SIRT1 Activity Assay Kit (Abcam) per manufacturer’s instructions with the following exceptions: 10 nM of Trichostatin A (Sigma) was added to each well to block histone deacetylase activity, ensuring specificity of SIRT1 activity and NAD^+^ was not added.

### Comet assay

Cells were plated at 5 × 10^5^ (SK-N-AS, BE(2)C) or 1 × 10^6^ (Kelly, SY5Y)/10-cm plate and treated 48 h after plating. Cells were detached with 0.05% trypsin (ThermoFisher), washed, and resuspended at 5 × 10^5^ cells/mL in cold PBS (ThermoFisher). DNA damage was analyzed using the CometAssay Single Cell Gel Electrophoresis Assay (Bio-Techne) following the Alkaline Comet Assay protocol per manufacturer’s instructions. Samples were viewed and imaged using a Nikon Eclipse TE300 microscope. At least six images of different frames per condition were captured. Comet analysis was conducted using OpenComet software tool for tail length and tail intensity.

### Annexin V-FITC propidium iodide assay

Cells were plated at 1–2 × 10^6^/10-cm plate overnight before treatment and collected at 24-, 48-, or 72 h using 0.25% trypsin (ThermoFisher), before counting and resuspension at 1 × 10^6^ cells/mL in PBS (ThermoFisher). Cells were processed following the Annexin V FITC Assay Kit protocol (Cayman Chemical) and flowed through CytoFLEX S Flow Cytometer (Beckman Coulter). Data analysis was done using FlowJo V.10.

### In vivo studies

NCI-Bethesda and NCI-Frederick are accredited by AAALAC International and follow the Public Health Service Policy for the Care and Use of Laboratory Animals. Animal care was provided in accordance with the procedures outlined in the ‘Guide for Care and Use of Laboratory Animals’ (National Research Council; 1996; National Academy Press; Washington, D.C.). All animal studies were approved by the NIH Animal Care and Use Committee. Four to six-week old female athymic nude mice (n = 8–14/condition) were purchased from Charles River Laboratories. Stable luciferase green fluorescent protein (GFP)-expressing cells were generated by lentivirus transduction and selected with 0.5 ug/mL of puromycin (Sigma). Luciferase GFP expression was confirmed by flow cytometry as previously described [38].

### Cell line orthotopic xenograft models

Orthotopic para-adrenal surgery was conducted for intra-adrenal injection of 3 × 10^5^ cells in Hanks’ Balanced Salt Solution in the right adrenal gland of each mouse as previously described [39]. All mice underwent weekly ultrasound (U/S) and bioluminescence imaging (BLI) for tumor monitoring and weekly body weight measurements. For both models, staggered enrollment of mice into groups was conducted when U/S tumor volume > 50 mm^3^ (SY5Y) or > 500 mm^3^ BE(2)C and mice were randomized to vehicle (30% 2- HPBCD and 1 M HCl) or OT-82 (50 mg/kg) which was administered by oral gavage using the clinical schedule of 3 days on/4 days off for 4 consecutive weeks. Mice were followed until humane endpoint, tumor endpoint (U/S maximum volume > 4,000 mm^3^), or death, whichever occurred first. In accordance with this policy, animals that reached tumor or humane endpoints were promptly euthanized, without exception.

### PDX-derived orthotopic xenograft models

Surgery for orthotopic implantation of 5 × 10^5^ cells in the left adrenal gland of each mouse was conducted as previously described [35]. Tumor burden was monitored using weekly BLI; body weight was measured weekly. Once each mouse exhibited BLI > 10^7^ photon/s/cm^2^/sr, mice were randomized and enrolled into vehicle or OT-82 treatment group and treated per the same dosing schedule as cell line xenograft studies. Mice were euthanized 8 weeks after enrollment or when they reached humane endpoint or death, whichever occurred first.

### Tumor monitoring studies

BLI and/or U/S imaging was used to monitor tumor growth for the duration of each study. Mice were anesthetized in the induction chamber with 3% isoflurane and injected with 150 mg/kg of d-luciferin potassium salt intraperitoneally before being transferred to imaging chamber with 2% isoflurane with O_2_ as a carrier at 1 L/minute flow rate. Animals from cell line xenograft studies were imaged on IVIS Spectrum (PerkinElmer). Animals from PDX-derived cell line xenograft studies were imaged using IVIS Lumina XR System (PerkinElmer). Living Image Software (PerkinElmer) was used to process imaging data via analysis of region of interest. U/S procedure has been previously described [40]. Standard imaging and animal handling procedures for imaging were followed including maintenance of internal temperature and monitoring of anesthesia administration.

### Pharmacodynamic studies

Early harvest of tumor was conducted 2 h after third dose of treatment (n = 3–8 mice/group) in each study. Tissue was snap frozen with liquid nitrogen (cell line models) or dry ice (PDX-derived model) for later processing. Tumors were sonicated on ice with ultrasonic homogenizer (4710 series; Cole-Parmer) and total NAD levels were measured using NAD^+^/NADH Assay Kit (Abcam) as previously described [23].

### Statistical analyses

Statistical significance between two groups was determined using a Welch’s t-test. p < 0.05 was considered significant.

## Results

### Neuroblastoma cell lines exhibit exquisite sensitivity to clinically relevant NAMPTis

**Fig. 1:**
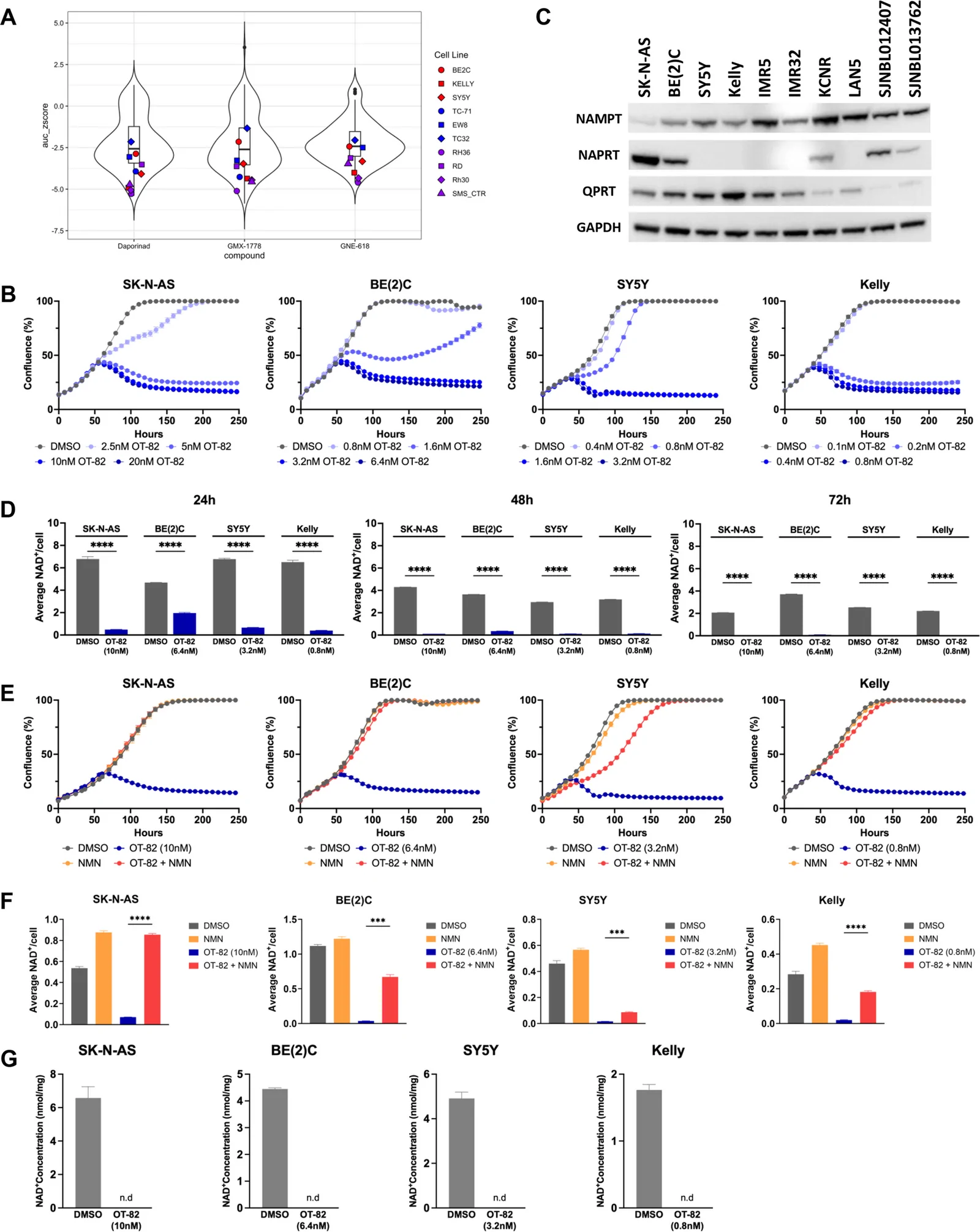
NB is highly susceptible to NAMPT inhibition which reduces cell proliferation and NAD^+^ levels. **A** High-throughput drug screening data displayed onto 3 violin plots comparing the activity of three NAMPTis (daporinad (*n* = 212), GMX-1778 (*n* = 212), and GNE-618 (*n* = 69) expressed as AUC Z-score. NB cell lines are highlighted in red; Ewing sarcoma cell lines are highlighted in blue; rhabdomyosarcoma cell lines are highlighted in purple. The box and whisker plot in the center of each violin represents the median (center line), the 25th to 75th percentile of the data (box), and the value of 1.5 × the interquartile range (whiskers). **B** IncuCyte live cell proliferation analysis of NB cell lines SK-N-AS (5 k/well), BE(2)C (5 k/well), SY5Y (10 k/well), and Kelly (10 k/well) treated with DMSO (gray) or varying doses of OT-82 (blue). Error bars represent SEM. Data shown is representative of one experiment. Each experiment was performed at least 3 times with at least 5 technical replicates. **C** Western blot of rate-limiting enzymes (NAMPT, NAPRT, and QPRT) in the three redundant NAD^+^ production pathways from whole cell extracts of a panel of 8 molecularly diverse NB cell line models and 2 PDX cell line models. GAPDH is loading control. Each experiment was performed at least 3 times. **D** Promega NAD/NADH-Glo Assay measuring average NAD^+^ levels per cell number in NB models at 24 h, 48 h, and 72 h after treatment with DMSO (gray) or OT-82 (SK-N-AS 10 nM, BE(2)C 6.4 nM, SY5Y 3.2 nM, and Kelly 0.8 nM) (blue). Error bars represent SEM. Each experiment was performed at least 3 times with at least 4 technical replicates. **E** IncuCyte live cell proliferation analysis of NB models treated with DMSO (gray), OT-82 (SK-N-AS 10 nM, BE(2)C 6.4 nM, SY5Y 3.2 nM, and Kelly 0.8 nM) (blue), 1 mM NMN (yellow), or co-treatment of OT-82 and NMN (red). Error bars represent SEM. Data shown is representative of one experiment. Each experiment was performed at least 3 times with at least 5 technical replicates. **F** Promega NAD/NADH-Glo Assay measuring average NAD^+^  levels per cell number in NB models at 72 h after treatment with DMSO (gray), OT-82 (SK-N-AS 10 nM, BE(2)C 6.4 nM, SY5Y 3.2 nM, and Kelly 0.8 nM) (blue), 1 mM NMN (yellow), or co-treatment of OT-82 and NMN (red). Error bars represent SEM. Each experiment was performed at least 3 times with at least 4 technical replicates. **G** NMR metabolomics analysis of intracellular NAD^+^ levels in NB models treated with DMSO (gray) or OT-82 (SK-N-AS 10 nM, BE(2)C 6.4 nM, SY5Y 3.2 nM, and Kelly 0.8 nM) (blue). Each experiment had 3 technical replicates. Error bars represent SD. n.d. = not detectable. Welch’s t-test was performed for each experiment. ****p < 0.0001; ***p < 0.001; **p < 0.01; *p < 0.05

Our lab and others have previously reported on the remarkable sensitivity of pediatric sarcomas EwS and RMS to NAMPTis [19–23]. Given the rarity of these entities and the dearth of novel therapies available for many pediatric malignancies, we sought to determine whether other pediatric solid tumors of unmet need might exhibit equivalent sensitivity to NAMPTis. To do so, we mined datasets generated at the National Center for Advancing Translational Sciences involving single agent cytotoxicity of the MIPE chemogenomics library of approved and investigational drugs versus a large panels of adult and pediatric cancer cell line models. Among > 200 cancer cell lines representing > 50 solid tumor types, two of the three NB cell lines in the screen (BE(2)C, SY5Y, and Kelly) exhibited high sensitivity to all three first-generation NAMPTis (daporinad, GMX-1778, and GNE-618) in the dataset, with IC_50_ values ~ 20-fold lower than the mean IC_50_ for the panel of cancer cell lines (Fig. 1A; Supplemental Fig. S1A). Furthermore, these NB cell lines were equally or more sensitive to the NAMPTis than RMS and EwS cell lines, respectively.

To validate and extend the results from the drug screen, we expanded our panel of NB models to include five additional cell lines, including one PDX-derived low passage cell line, selected to represent a range of molecular features relevant to NB biology, including *MYCN* amplification status, *ALK* mutation status, and *TP53* mutation status (Supplemental Table S2). Using longitudinal live-cell image analysis, we tested the effects of three NAMPTis: first-generation clinical molecule daporinad and two second-generation clinical molecules OT-82 and KPT-9274 (Fig. 1B; Supplemental Fig. S1B-D). We calculated IC_50_ values using the longitudinal assay data, determining the concentration of OT-82 that inhibited 50% of cell growth at the timepoint when the control cells reached 100% confluence. In all NB models tested, we observed an across-the-class dose-dependent antiproliferative effect of all three NAMPTis, with a range of active doses and IC_50_ values depending on the model and NAMPTi (0.1–5 nM for OT-82; 0.2–6.4 nM for daporinad; 4–1000 nM for KPT-9274) (Supplemental Table S3).

As one potential biomarker of NAMPTi sensitivity in cancer is low or absent expression of compensatory NAD^+^ production enzymes [9, 41–47], we performed immunoblot analysis of NB cell lines to determine expression of rate-limiting enzymes in the three redundant NAD^+^ production pathways: NAMPT, NAPRT, and QPRT. While universal expression of NAMPT and QPRT were confirmed across all NB models, albeit variably expressed, NAPRT expression was undetectable in five of ten models (Fig. 1C). In this panel, three of the four NB cell lines with the highest expression of NAPRT (SK-N-AS, BE-2C, and SJNBL012407) exhibited the highest IC_50_, and formal analysis of the full cell line panel revealed a correlation between NAPRT expression and IC_50_ (r = 0.9723, p < 0.0001, (95%CI [0.87,0.99]). Although NAPRT expression does not preclude sensitivity to NAMPTis, this finding may suggest that NAPRT deficiency contributes to a differential in NAMPTi sensitivity between NAPRT expressing and non-expressing NB cell lines. Based on this data, we selected a subset of four cell lines (SK-N-AS, BE(2)C, Kelly, and SY5Y) in which to carry out additional experiments, curated to include characteristics relevant to NAD^+^ production and prognostic NB disease biology, namely NAPRT expression and *MYCN* amplification status (Supplemental Table S2), with two *MYCN* amplified cell lines (BE(2)C and Kelly) and two NAPRT-deficient cell lines (Kelly and SY5Y) in the panel.

### Clinical NAMPTis reduce NAD^+^ and NADH in NB cell line models

To investigate effects of the NAMPTis on the putative target, we measured intracellular NAD^+^ and NADH levels after treatment with the clinically relevant NAMPTis: OT-82 and KPT-9274. Using the lowest doses of each NAMPTi that induced complete growth inhibition in each cell line, we observed a time-dependent reduction in NAD^+^ levels between 24 and 72 h compared to control cells (Fig. 1D; Supplemental Fig. S2A). NADH levels in treated cells were similarly reduced (Supplemental Fig. S2B-C). To verify that loss of NAD^+^ and NADH was due to decreased NAMPT activity, we used nicotinamide mononucleotide (NMN), the product of NAMPT, in co-treatment experiments with OT-82 and KPT-9274 and observed rescue of cell proliferation in all models (Fig. 1E; Supplemental Fig. S2D). Consistent with these findings, analysis of NAD^+^ levels in these conditions demonstrated that NAD^+^ levels were also at least partially rescued with NMN co-treatment (Fig. 1F; Supplemental Fig. S2E), confirming on-target effects of OT-82 and KPT-9274 in NB cells.

As a second confirmation of on-target activity, we directly measured intracellular NAD^+^ and NADH levels with NMR and mass spectrometry, respectively, and confirmed that OT-82 significantly reduced NAD^+^ and NADH levels at 24 h in all four models compared to control-treated cells (Fig. 1G; Supplemental Fig. S2F-G). As KPT-9274 is known to target both NAMPT and PAK4, we chose to proceed with further mechanistic analyses in the four NB tumor cell lines using OT-82, as this agent is a sensitive and specific inhibitor of NAMPT with no known additional molecular targets.

### Loss of NAD^+^ by NAMPT inhibition significantly impacts energy metabolism in NB models

**Fig. 2:**
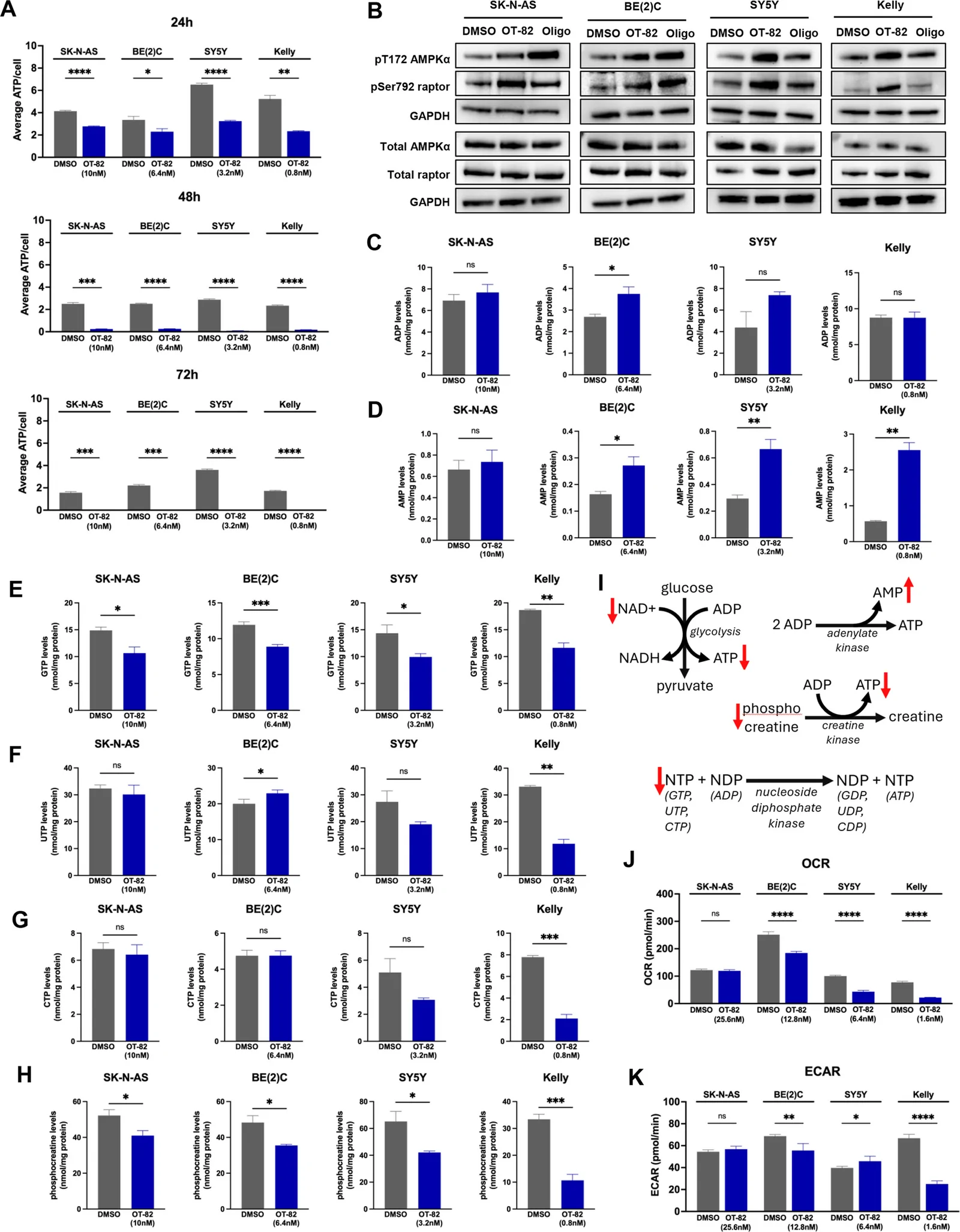
Energy metabolism is significantly impacted by loss of NAD^+^ in NB. **A** Cell Titer-Glo assay measuring ATP production normalized to cell number in NB models treated with DMSO (gray) or OT-82 (SK-N-AS 10 nM, BE(2)C 6.4 nM, SY5Y 3.2 nM, and Kelly 0.8 nM) (blue) at 24 h, 48 h, and 72 h. Each experiment was performed at least 3 times with at least 4 technical replicates. **B** Western blot showing pT172 AMPK, pSer792 raptor, total AMPK, and total raptor in NB cells treated with DMSO, OT-82 (SK-N-AS 10 nM, BE(2)C 6.4 nM, SY5Y 3.2 nM, and Kelly 0.8 nM) (blue) for 24 h, or positive control oligomycin 10 uM for 1 h. GAPDH is loading control. Each experiment was performed at least 2 times. **C-H** ICMS analysis of (**C**) ADP, (**D**) AMP, (**E**) GTP, (**F**) UTP, (**G**) CTP, and (**H**) phosphocreatine levels in NB models treated with DMSO (gray) or OT-82 (SK-N-AS 10 nM, BE(2)C 6.4 nM, SY5Y 3.2 nM, and Kelly 0.8 nM) (blue) at 24 h. Each experiment had 3 technical replicates. Error bars represent SD. **I** Schematic diagram illustrating impaired cytosolic ATP production caused by NAD^+^ depletion and resulting in compensatory nucleoside phosphoryl transfer reactions including creatine kinase, adenylate kinase, and nucleoside diphosphate kinase enzymatic reactions. **J** Basal OCR (pmol/minute) measured in NB models following treatment with DMSO (gray) or OT-82 (SK-N-AS 25.6 nM, BE (2)C 12.8 nM, SY5Y 6.4 nM, Kelly 1.6 nM) (blue) for 24 h. Each experiment was performed at least 3 times with at least 5 technical replicates. **K** Glycolytic rate measured by extracellular acidification rate (ECAR) measured in NB models following treatment with DMSO (gray) or OT-82 (SK-N-AS 25.6 nM, BE(2)C 12.8 nM, SY5Y 6.4 nM, Kelly 1.6 nM) (blue) for 24 h. Each experiment was performed at least 3 times with at least 5 technical replicates. Welch’s t-test was performed for each experiment. ****p < 0.0001; ***p < 0.001; **p < 0.01; *p < 0.05

As the downstream mechanistic effects of NAD^+^ loss are diverse and may be cell-type specific, we next examined transcriptomic changes induced by OT-82 in NB cell lines to identify affected pathways. We again used the lowest concentrations of OT-82 that induced complete growth inhibition in each cell line and treated cells for 24 h, a timepoint where we observed on-target depletion of NAD^+^ but minimal effect on proliferation. Using KEGG pathway enrichment analysis of differentially expressed genes from each of the four treated and untreated cell lines, we identified that OT-82 treatment was associated with upregulation of genes in pathways related to energy metabolism, including oxidative phosphorylation (OXPHOS) and carbon metabolism (Supplemental Fig. S3). To further interrogate this finding, we investigated production of ATP after OT-82 treatment in NB cell lines. In cell viability assays using ATP quantitation as an indicator of metabolically active live cells, we observed that within 24 h of drug exposure, OT-82 induced dose- and time-dependent reductions in NB cell viability/ATP levels at the same doses that mediated eventual loss of proliferation in longitudinal growth assays (Fig. 2A). Reduction in ATP levels was associated with induction of phospho-AMPK and phospho-raptor in all cell lines after 24 h of OT-82, signifying energy deprivation (Fig. 2B; Supplemental Figure S4A). As an additional consequence of energy stress, we observed significant accumulation of either ADP, AMP or both, in three of four cell lines (Fig. 2C-D). Furthermore, analysis of cellular energy charge revealed a statistically significant decrease in ATP/ADP ratios in two of four cell lines and a decrease in ATP/AMP ratios in three of four cell lines at 24 h (Supplemental Figure S4B-C). We also observed a significant decrease in GTP levels in all four cell lines (Fig. 2E) whereas UTP and CTP levels were decreased only in SY5Y and Kelly following OT-82 treatment (Fig. 2F-G), suggesting there may be a differential effect on phosphorylation status of purine versus pyrimidine nucleotides that may correlate with drug sensitivity. Finally, phosphocreatine levels were significantly decreased in all four cell lines (Fig. 2H), demonstrating a reduction in reserve cellular phosphorylation power resulting from OT-82 mediated NAD^+^ depletion (Fig. 2I). Together these data are consistent with impaired cytosolic ATP production caused by NAD^+^ depletion and resulting in compensatory nucleoside phosphoryl transfer reactions likely including creatine kinases, adenylate kinases, and nucleoside diphosphate kinases (Fig. 2I).

Cellular ATP is generated during both glycolysis and OXPHOS, and NAD^+^ is necessary for both processes acting as a soluble electron transfer intermediate. To distinguish between the effects of NAD^+^ depletion on glycolysis and OXPHOS, we used extracellular flux analysis to detect changes in OXPHOS via measurement of OCR and lactate fermentation via measurement of ECAR. Upon OT-82 exposure, OCR was significantly reduced in all NB cell lines except SK-N-AS, suggesting that OXPHOS impairment is a common downstream consequence of NAD^+^ depletion in NB cells (Fig. 2J). In contrast, ECAR was significantly reduced in only two of four NB cell lines (BE (2)C and Kelly), remaining unchanged in SK-N-AS and increasing in SY5Y upon OT-82 treatment (Fig. 2K). Amperometric measurement of glucose concentrations in culture media supernatants demonstrated that a subset of cell lines exhibited diminished glucose consumption following 72 h treatment with OT-82 (Supplemental Figure S4D). Taken together, these results suggest that the effects of NAD^+^ loss on glucose utilization and oxidative phosphorylation in NB cells may be cell line specific.

#### Alterations in metabolite levels reveal specific pathways targeted by NAD^+^ depletion

**Fig. 3:**
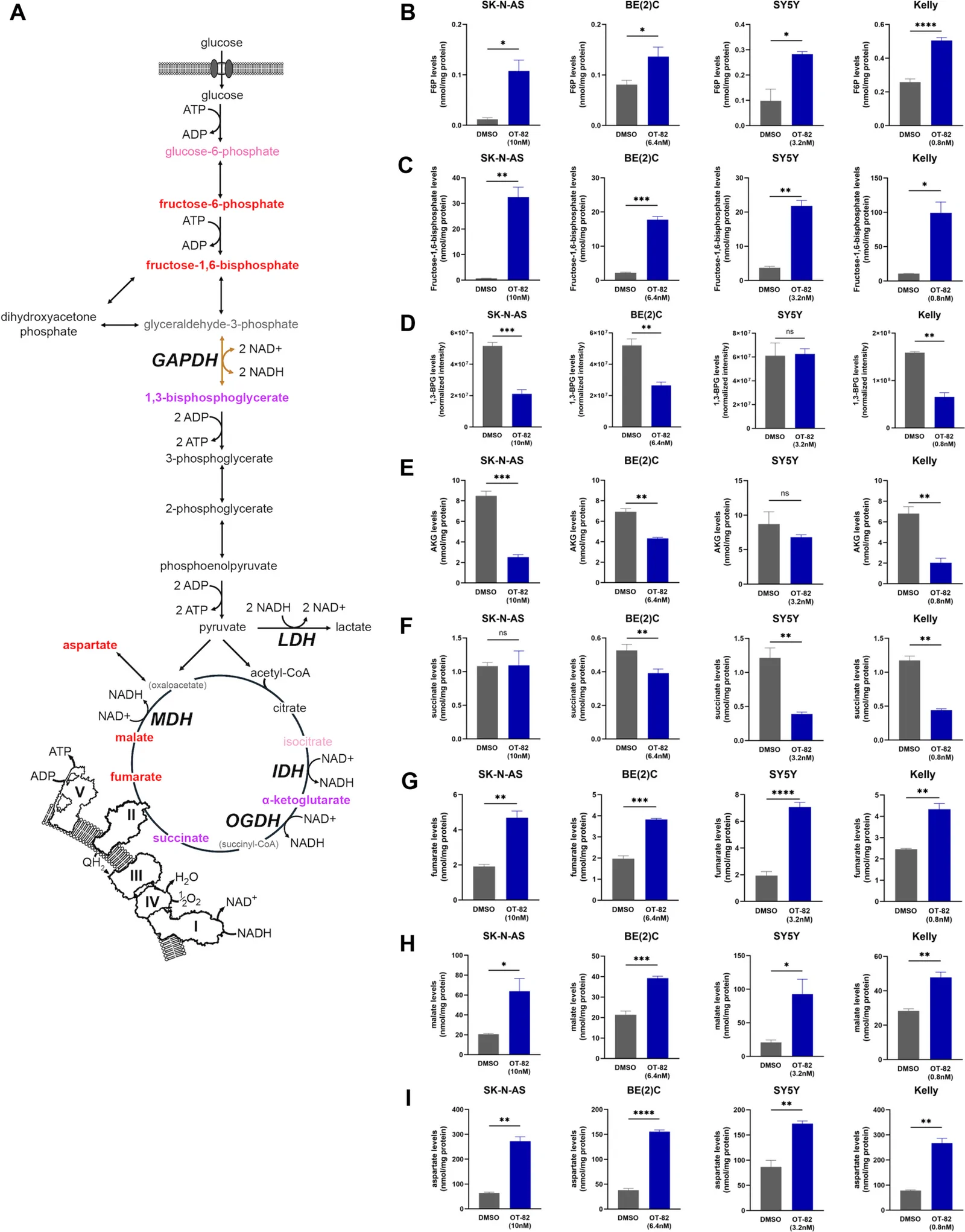
OT-82 impacts glycolysis and TCA cycle metabolites. **A** Glycolysis and TCA cycle metabolite pathway summary maps showing key changes in metabolite concentration following OT-82 treatment at 24 h measured by ICMS. Increase in metabolite concentration following OT-82 treatment of 2/4 cell lines are highlighted in pink and for > 2/4 cell lines in red. Decrease in metabolite concentration following OT-82 treatment for > 2/4 cell lines are highlighted in purple. NAD^+^-dependent enzymes are notated in bold italic: GAPDH: glyceraldehyde-3-phosphate dehydrogenase; LDH: lactate dehydrogenase; IDH: isocitrate dehydrogenase; OGDH: 2-oxoglutarate dehydrogenase; MDH: malate dehydrogenase. ICMS analysis of **B** fructose-6-phosphate, **C** fructose-1,6-bisphosphate, **D** 1,3-bisphosphoglycerate, **E** α-ketoglutarate, **F** succinate, **G** fumarate, **H** malate, and **I** aspartate metabolite levels in NB models treated with DMSO (gray) or OT-82 (SK-N-AS 10 nM, BE(2)C 6.4 nM, SY5Y 3.2 nM, and Kelly 0.8 nM) (blue) at 24 h. Each experiment had 3 technical replicates. Error bars represent SD. Welch’s t-test was performed for each experiment. ****p < 0.0001; ***p < 0.001; **p < 0.01; *p < 0.05

To further investigate the mechanistic basis by which NAMPT inhibition affects central metabolic pathways, we quantified specific glycolysis and TCA cycle intermediate metabolites in untreated and OT-82 treated cells, using the same 24 h time point and same concentrations of OT-82 as we used for the transcriptomic analysis. Glycolytic flux relies on abundant levels of oxidized NAD^+^ to serve as the electron acceptor for the glyceraldehyde-3-phosphate dehydrogenase (GAPDH) reaction which allows glucose-derived carbon to proceed to the “payoff phase” of glycolysis forming a net of two ATP molecules. Using targeted ICMS analysis, we quantified changes in glycolytic intermediates in OT-82-treated cells (Fig. 3A-D; Supplemental Fig S4E-F) and found that upon OT-82 treatment, glucose-6-phosphate levels were elevated in two of four cell lines (Supplemental Fig. S4E), whereas the downstream intermediate fructose-6-phosphate (F6P) was increased in all four cell lines (Fig. 3B). Levels of fructose-1,6-bisphosphate (FBP) were increased in all four cell lines (Fig. 3C). In contrast, levels of the product of the GAPDH reaction 1,3-bisphosphoglycerate (1,3-BPG) were decreased in three of four cell lines (Fig. 3D). Together, these results suggest profound inhibition of glycolysis at the level of the GAPDH reaction.

While glycolysis relies on the continuing supply of cytosolic NAD^+^ for the GAPDH reaction, several enzymatic steps of the TCA cycle are also NAD^+^-dependent. The isocitrate dehydrogenase reaction which occurs in both the cytosol and mitochondria utilizes NAD(P)^+^ to decarboxylate isocitrate to form α-ketoglutarate and CO_2_. Levels of isocitrate were increased in two of four OT-82-treated NB cell lines (Supplemental Fig. S4F), while α-ketoglutarate was significantly decreased in three of four cell lines (Fig. 3E). Notably, succinate levels were also decreased in three of four cell lines (Fig. 3F), while fumarate and malate levels increased in all cell lines (Fig. 3G-H). Finally, aspartate levels in all OT-82 treated cell lines rose significantly (Fig. 3I), suggesting either decreased aspartate utilization and/or increased aspartate production. Together, these data suggest that multiple NAD^+^-dependent TCA cycle enzymatic reactions are modulated following NAD^+^ depletion.

### NAMPT inhibition impairs activity and function of NAD^+^ consuming enzymes: sirtuin-1 and poly (ADP-ribose) polymerase (PARP) and induces DNA damage in NB models

**Fig. 4:**
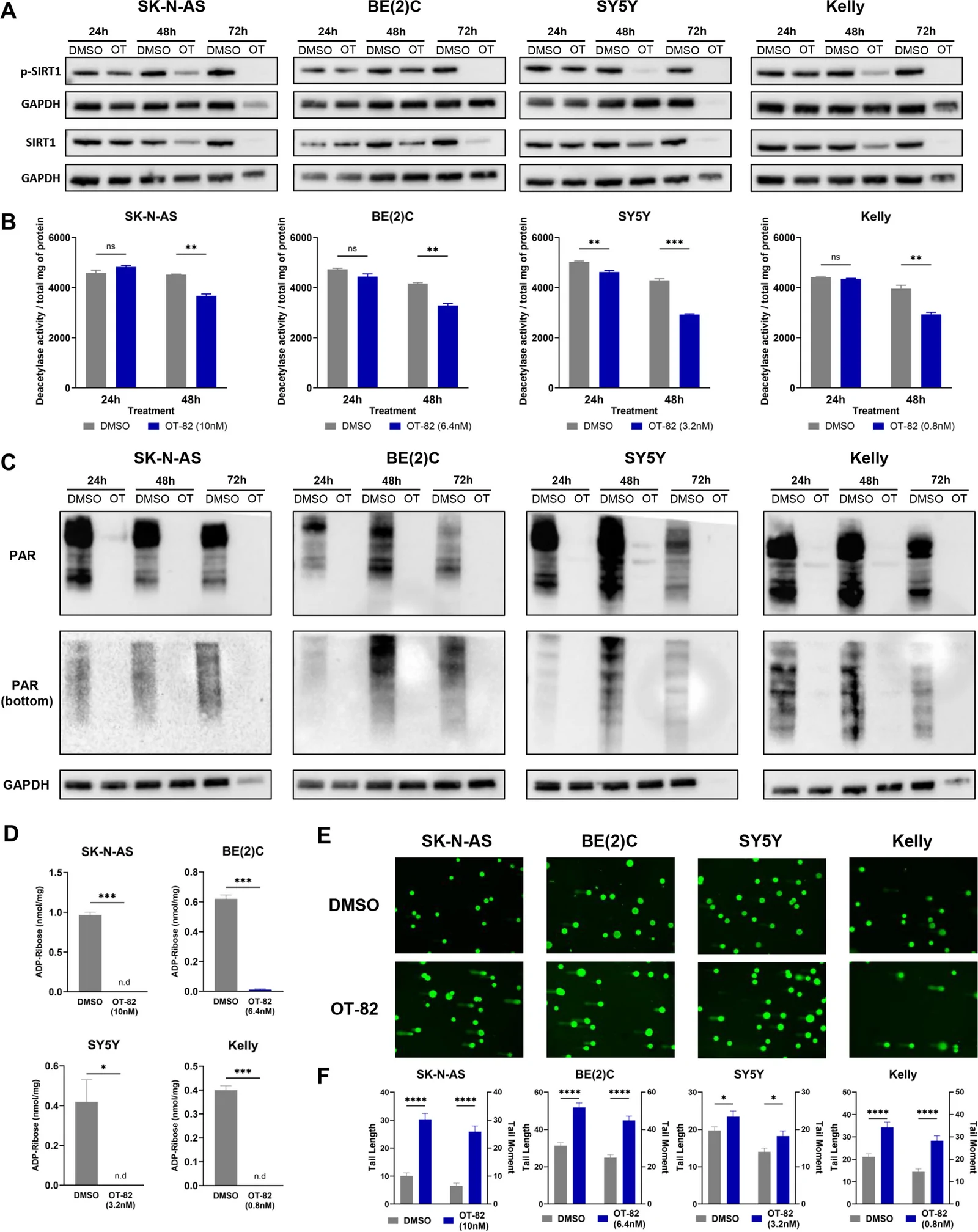
Functional activity of NAD^+^-dependent substrates SIRT1 and PARP is significantly reduced with OT-82 treatment. **A** Western blot analysis of phosphorylated-SIRT1 (p-SIRT1) and total SIRT1 protein expression of whole cell extracts of NB cells following treatment with DMSO or OT-82 (SK-N-AS 10 nM, BE(2)C 6.4 nM, SY5Y 3.2 nM, and Kelly 0.8 nM) at 24 h, 48 h, and 72 h. GAPDH is loading control. Each experiment was performed at least 3 times. **B** Sirtuin activity assay normalized to protein in NB cells at 24 h and 48 h treated with DMSO (gray) or OT-82 (SK-N-AS 10 nM, BE(2)C 6.4 nM, SY5Y 3.2 nM, and Kelly 0.8 nM) (blue). Each experiment was performed at least 3 times with at least 4 technical replicates. (**C**) Western blot analysis of PAR protein expression from whole cell extracts of NB cells at 24 h, 48 h, and 72 h treated with DMSO or OT-82 (SK-N-AS 10 nM, BE(2)C 6.4 nM, SY5Y 3.2 nM, and Kelly 0.8 nM). GAPDH is loading control. Each experiment was performed at least 3 times. (**D**) ICMS analysis of ADP ribose in NB models treated with DMSO (gray) or OT-82 (SK-N-AS 10 nM, BE(2)C 6.4 nM, SY5Y 3.2 nM, and Kelly 0.8 nM) (blue) at 24 h. Each experiment had 3 technical replicates. Error bars represent SD. Welch’s t-test was performed for each experiment. ***p < 0.001; *p < 0.05. **E** Representative comet assay images of NB cell lines treated for 24 h with DMSO or OT-82 (SK-N-AS 10 nM, BE(2)C 6.4 nM, SY5Y 3.2 nM, and Kelly 0.8 nM). Each experiment was performed at least 3 times with at least 2 technical replicates. (**F**) Quantification tail length and tail moment from full set of comet images (6–12 technical replicates) collected from the control and treatment groups over three separate biological replicate experiments

Outside of its role in energy metabolism, NAD^+^ is a crucial co-substrate for sirtuins and poly (ADP-ribose) polymerase (PARP) proteins. As PARP proteins and sirtuins compete for the same limited NAD^+^ pool, PARP activation (e.g. by DNA damage or oxidative stress) may reduce sirtuin activity whereas PARP inhibition can boost sirtuin activity [48]. We determined whether treatment with OT-82 affects the abundance and activity of SIRT1, the main nuclear sirtuin. We first measured the levels of the active form of SIRT1, phosphorylated on Threonine 530 (p-SIRT1). This phosphorylation activates SIRT1 and is essential to prevent DNA breakage and maintain genomic stability [49]. We observed reductions of p-SIRT1 at 48 h in three models and in all four models by 72 h. Full length SIRT1 also decreased beginning at 48 h of treatment (Fig. 4A**;** Supplemental Fig. S5A). To determine the functional effect on sirtuin activity, we next used a SIRT1 activity assay to measure SIRT1 activity kinetics in the presence of OT-82. Consistent with the phosphorylation result, we observed a decrease in SIRT1 activity after 48 h of OT-82 treatment (Fig. 4B).

To determine the effects of OT-82 on PARP activity, we performed immunoblot analysis of OT-82 treated cells to measure ADP-ribosylated protein (PAR) expression as ADP-ribosylation is a post-translational modification that is catalyzed by PARPs. We observed reductions of PAR expression in all four cell lines beginning at 24 h post treatment (Fig. 4C; Supplemental Fig. S5B). The reduction in ADP-ribosylation of proteins was also associated with a profound decrease in soluble ADP-ribose, which fell to undetectable levels in all NB cell lines except BE(2)C (Fig. 4D). As loss of sirtuin 1 and PARP1 activity can compromise DNA repair, we next investigated whether NAD^+^ depletion by OT-82 results in DNA damage accumulation via comet assay. We observed an increase in comet tails in all four cell lines after 24 h of OT-82 treatment (Fig. 4E-F). Together, these data demonstrate that OT-82 treatment impacts the activity of multiple downstream NAD^+^-dependent processes, resulting in DNA damage in NB models.

### NAMPT inhibition induces non-apoptotic cell death in NB cell lines

**Fig. 5:**
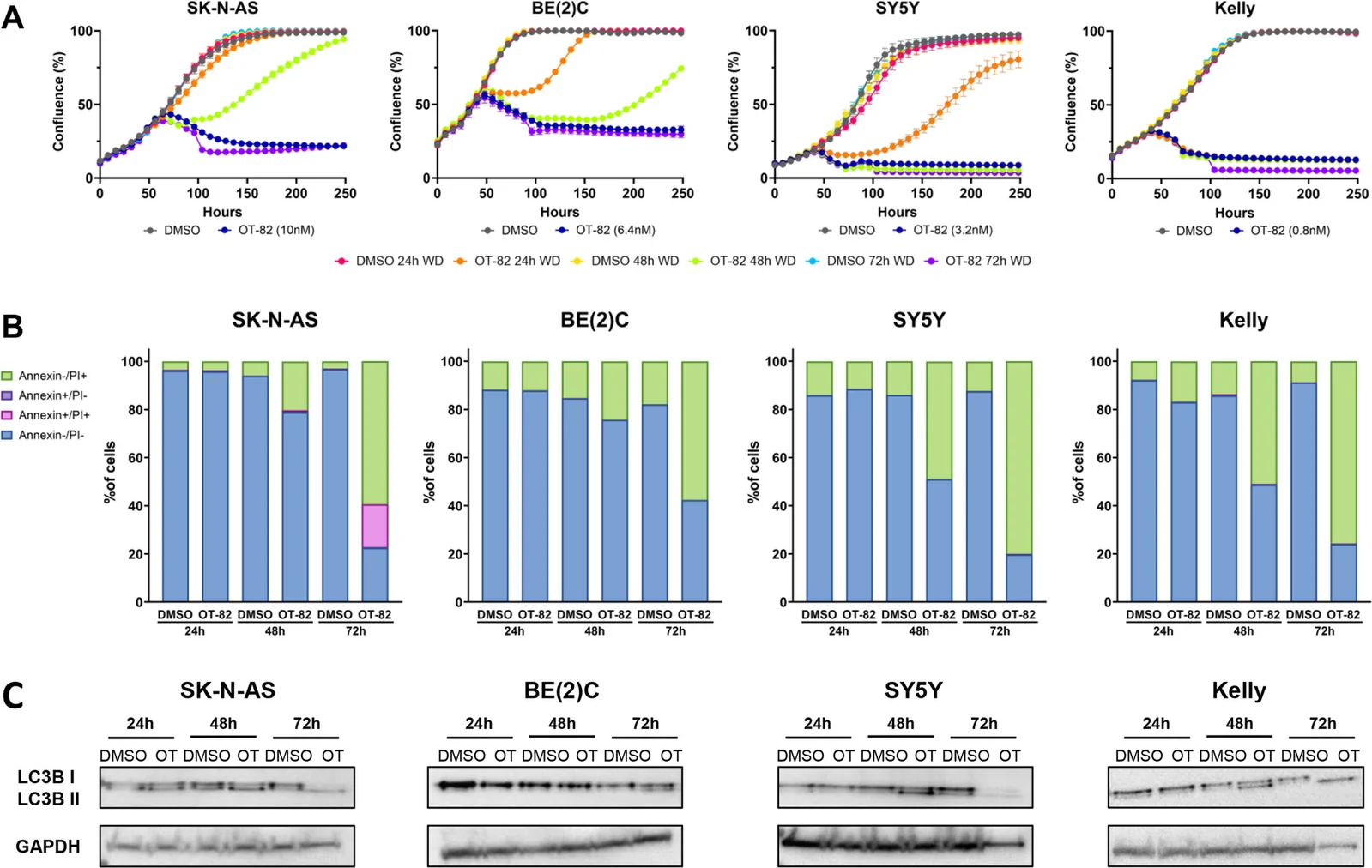
NB cells undergo irreversible non-apoptotic cell death. **A** IncuCyte live cell proliferation analysis of NB cell lines SK-N-AS (5 k/well), BE(2)C (5 k/well), SY5Y (10 k/well), Kelly (10 k/well) treated with DMSO or OT-82 with replacement with drug-free media at 24 h, 48 h, and 72 h. Error bars represent SEM. Data shown is representative of one experiment. Each experiment was performed at least 3 times with at least 5 technical replicates. **B** Annexin/PI analysis of NB cell lines at 24 h, 48 h, and 72 h treated with DMSO or OT-82. Annexin-/PI- represents live cells population (blue), Annexin +/PI + represents late apoptotic cells (pink), Annexin +/PI- represents early apoptotic cells (purple), and Annexin-/PI + represents necrotic cells (green). Each experiment was performed at least 3 times with at least 2 technical replicates. **C** Western blot analysis of LC3-I and LC3-II from whole cell extracts of NB cells treated for 24 h, 48 h, and 72 h with DMSO or OT-82. GAPDH is loading control. Each experiment was performed at least 3 times

To assess the durability of the antiproliferative responses we observed, we replaced drug media with drug-free media after three different exposure times (24-, 48-, and 72 h) and observed that within 72 h of OT-82 exposure, replacement with drug-free media failed to rescue proliferation in all cell lines, suggesting that an irreversible block in proliferation or irreversible cell death occurs upon prolonged drug exposure (Fig. 5A). To further explore this, we first characterized the effects of OT-82 on the cell cycle and did not observe evidence of cell cycle arrest in any of the cell lines at any of the timepoints (Supplemental Fig. S6A). Similarly, examination of the morphology of OT-82 treated cells at multiple timepoints did not suggest cellular differentiation was occurring (Supplemental Fig. S6B). Next, we examined transcriptomic data and surprisingly did not observe enrichment in genes associated with the apoptotic pathway (Supplemental Fig. S6C). We confirmed these findings using Annexin V/propidium iodide (PI) staining of NB cells treated with OT-82 at 24-, 48-, and 72 h. In all models, we observed that OT-82 treatment was associated with an increase in the proportion of cells stained positive for PI only beginning at 48 h. However, double staining with PI and Annexin V was only observed in one cell line and occurred 24 h after the induction of PI staining alone, suggesting that OT-82 treatment results in a non-apoptotic mechanism of cell death (Fig. 5B; Supplemental Fig. S6D). To further characterize this non-apoptotic phenotype, we evaluated necroptosis pathway proteins; immunoblot analysis did not reveal phosphorylation of necroptosis markers RIPK1 or MLKL in any NB cell lines, suggesting that necroptosis is not a primary mechanism of cell death in NB (Supplemental Fig. S6E). As autophagy has been reported as a mechanism of NAMPTi -mediated cell death in other cancer types, [50, 51] we next examined protein levels of autophagy markers. We observed an enhancement of LC3-II expression in each cell line (Fig. 5C). However, this occurred between 48 and 72 h of OT-82 treatment, suggesting that OT-82 is unlikely to be a primary autophagy inducer but rather that initiation of autophagy is a secondary or compensatory cellular response to NAD^+^ depletion in NB cells. Similarly, OT-82 treatment failed to elevate ROS levels, suggesting that ROS-mediated oxidative stress is not a major contributor to the observed cell death (Supplemental Fig. S6F).

### In vivo models of NB exhibit tumor regression with NAMPT inhibition

**Fig. 6:**
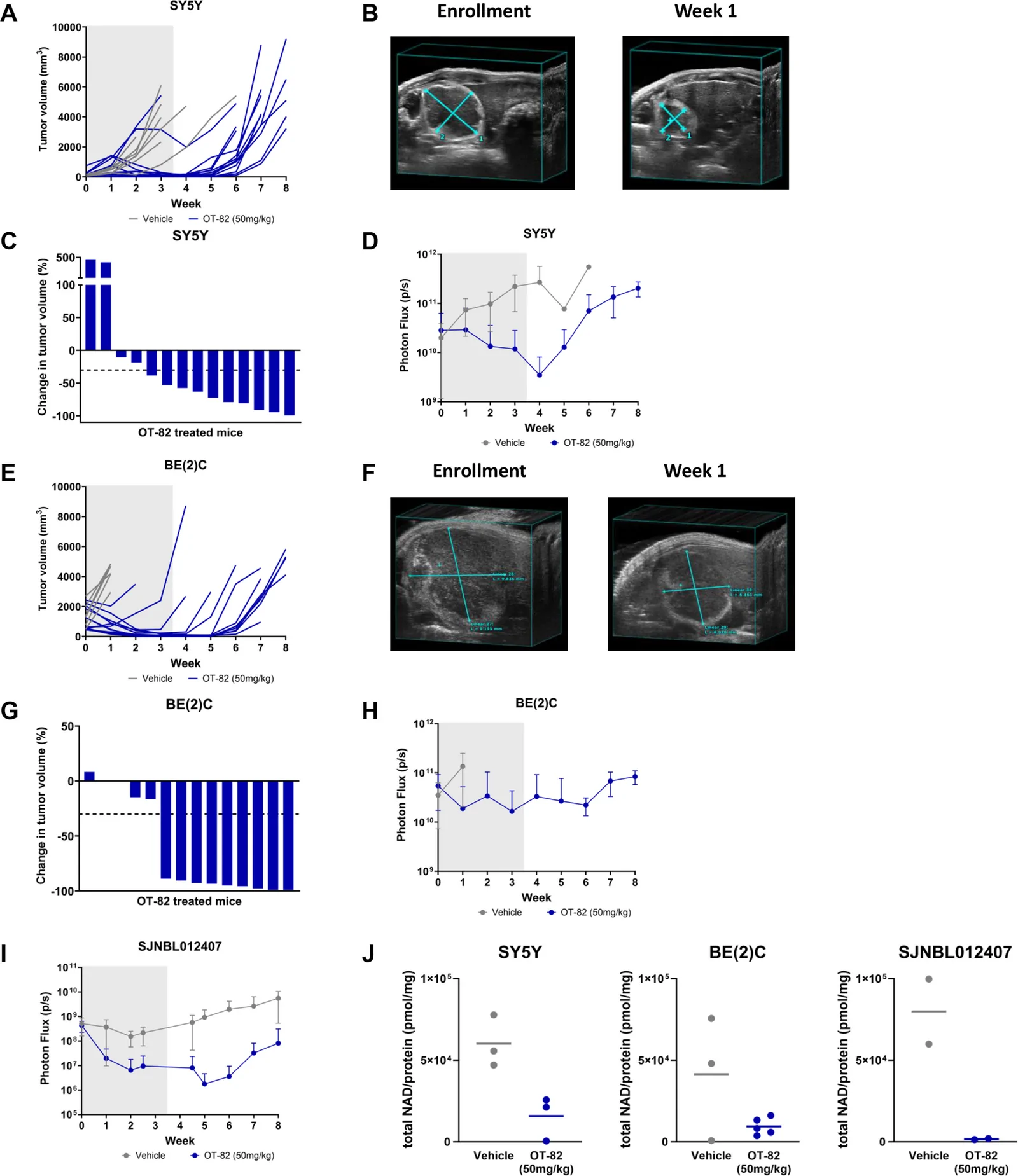
In vivo use of OT-82 in orthotopic models of NB demonstrates clinical benefit. **A** Tumor volume measured by ultrasound imaging of SY5Y-bearing xenografts in vehicle group (*n* = 8) (gray) or OT-82 treatment group (*n* = 14) (blue). Each line is representative of one mouse. Gray box indicates on treatment time. **B** Representative ultrasound images of one SY5Y mouse model at enrollment (left) and one week after OT-82 treatment (right). **C** Waterfall plot showing best tumor response in SY5Y-bearing xenograft model. Dotted line represents partial response defined as at least 30% regression. **D** Average photon flux measured by bioluminescence intensity imaging of SY5Y-bearing xenografts in vehicle group (*n* = 8) (gray) or OT-82 treatment group (*n* = 14) (blue) **E** Tumor volume measured by ultrasound imaging of BE(2)C-bearing xenografts in vehicle group (n = 8) (gray) or OT-82 treatment group (*n* = 14) (blue). Each line is one mouse. Gray box indicates on treatment time. **F** Representative ultrasound images of one BE(2)C mouse model at enrollment (left) and one week after OT-82 treatment (right). **G** Waterfall plot showing best tumor response in BE(2)C-bearing xenograft model. Dotted line represents partial response defined as at least 30% regression. **H** Average photon flux measured by BLI imaging of BE(2)C model of mice in vehicle group (*n* = 8) (gray) or OT-82 treatment group (*n* = 14) (blue). Gray box indicates on treatment time. **I** Average photon flux measured by BLI imaging of SJNBL012407 PDX mouse model in vehicle group (*n* = 9) (gray) or OT-82 treatment group (n = 10) (blue). Gray box indicates on treatment time. **J** Total NAD levels measured in tumor tissue from all three models

To determine in vivo efficacy of NAMPT inhibition in neuroblastoma, we evaluated OT-82 in three orthotopic NB models. For each model, OT-82 was administered on the clinical schedule of 3 days on/4 days off at 50 mg/kg for 4 weeks. For the first two models, both weekly BLI and U/S imaging were conducted to determine concordance of BLI signal and U/S-measured tumor volume. In the first study, the effects of OT-82 were tested in mice bearing SY5Y xenografts, using a traditional tumor volume of > 50 mm^3^ enrollment. OT-82 treatment resulted in stabilization or shrinkage of tumors in most OT-82-treated mice during the treatment period (Fig. 6A-B), with 10 of 14 mice bearing SY5Y xenografts, experiencing partial responses, defined by a tumor size decrease of at least 30% (range 38% to 99%) (Fig. 6C). Correspondingly, OT-82 treatment resulted in continuous decrease in average BLI signal during treatment (Fig. 6D) and a modest but statistically significant survival advantage in the treatment group compared to control (Supplemental Fig. S7A; Supplemental Dataset S1).

To more closely mimic the clinical scenario in patients, we next sought to investigate the effects of OT-82 when delaying the initiation of treatment until tumor size was larger (> 500 mm^3^). For this experiment, we used BE(2)C, a *MYCN* amplified and NAPRT proficient model which we expected to be less sensitive to NAMPT inhibition based on our in vitro experiments. Despite this, we observed similar tumor regression by U/S in most mice treated with OT-82 (Fig. 6E-F), with partial responses achieved in 9 of 14 mice bearing BE(2)C xenografts in the OT-82 treatment group (range 88% to 99%) (Fig. 6G). Again, a corresponding decrease in average BLI intensity was associated with decreased tumor volume by U/S while on treatment (Fig. 6H). We observed a more pronounced survival advantage in treated mice than in the prior model (Supplemental Fig. S7B; Supplemental Dataset S2).

In both studies, U/S imaging prior to treatment start revealed the presence of non-adrenal tumors, possibly representing regional metastases, in a subset of mice (8 of 22 mice bearing SY5Y xenografts and 7 of 22 mice bearing BE(2)C xenografts) which were indistinguishable from the primary adrenal tumor by BLI and were located predominantly in the liver. In both models, these regional metastases responded similarly to OT-82 as the primary tumors, stabilizing or shrinking in SY5Y xenografts (Supplemental Fig. S7C; Supplemental Dataset S1) and regressing in BE(2)C xenografts (Supplemental Fig. S7D; Supplemental Dataset S2). In both studies, we observed tumor recurrence approximately 1–3 weeks following cessation of treatment, indicated by rising BLI signal and U/S tumor volumes (Fig. 6A, E).

Since the previous studies suggested concordance between U/S measurements and BLI, we followed mice in third study with BLI alone, using a *MCYN* amplified, NAPRT proficient PDX-derived cell line (SJNBL012407). OT-82 treatment in this model demonstrated a decrease in BLI compared to vehicle-treated mice consistent with the prior two experiments (Fig. 6I). Throughout all studies, mice maintained their body weights and exhibited no drug-related toxicities (Supplemental Fig. S7E-G; Supplemental Dataset S3).

Pharmacodynamic analysis of total NAD in tissues harvested on day 3 of treatment demonstrated NAD depletion in the tumors of OT-82 treated mice compared to tumors of the vehicle treated mice in all three models, recapitulating the drug mechanism in vivo (Fig. 6J). In addition, tissue staining performed on material collected on day 3 of treatment demonstrated a trend towards increased tumor necrosis in the treated tumors even at this very early timepoint (Supplemental Fig. S7H). Together, these in vivo findings recapitulate the in vitro activity and primary mechanistic effects of OT-82 in multiple heterogeneous models of NB.

## Discussion

New treatments for high-risk NB are critically needed [52]. In this study, we identified that NB models are highly sensitive to NAMPTis, undergoing cell death at concentrations previously shown to be effective for other highly NAMPTi-sensitive models, including blood cancers, EWS, and RMS, and we conclude that NAMPTis may represent an effective therapeutic strategy against this disease. We are, to our knowledge, the first to demonstrate that NB is among the most NAMPTi-sensitive tumor types, a finding based on results from our unbiased large-scale cell line screen that showed that NAMPTi activity against NB models far exceeds that observed against other solid tumor models, with IC_50_ values approximately 20-fold lower for NB compared to those of over 200 other cancer cell lines. This observation provided the rationale for deeper translational investigation of NAMPT inhibition in NB.

Functionally, we have shown that multiple agents in the class impair proliferation and viability in an on-target manner across a molecularly diverse panel of NB cell lines. Mechanistically, NAMPT inhibition results in a cascade of deleterious effects, beginning with a marked decrease in both NAD^+^ and PARP activity that leads to the perturbation of energy metabolism at NAD^+^-dependent steps and the initiation of DNA damage accumulation by 24 h. By 48 h, cells enter a state of maximum energy stress, characterized by depletion of ATP, which triggers the initiation of autophagy as the cells attempt to maintain homeostasis. Concurrently, sirtuin loss is observed, leading to additional irreparable DNA damage. By 72 h, irreversible cell death occurs. Importantly, we further show that in vivo use of the clinically relevant NAMPTi OT-82 on the clinical dosing schedule results in tumor regressions in multiple orthotopic NB models designed to mimic the human disease, including models with large primary tumors and regional metastases. Our findings support those of a previous report demonstrating in vivo sensitivity of a single NB cell line flank model (NB1691) to the tool compound NAMPTi STF-118804 [53], and another demonstrating in vivo sensitivity of a different non-orthotopic NB cell line model (IMR-32) to the first generation NAMPTi GMX-1777 [54]. In contrast to these prior studies, which described NB cells undergoing apoptotic cell death when treated with STF-118804 or GMX-1777 [53, 54], we observed that OT-82 induced non-apoptotic cell death, suggesting that distinct NAMPTis or different doses of NAMPTis may engage different cell death pathways in NB cells.

While the precise reason for the enhanced sensitivity of NB is unknown, several potential explanations exist. The absence of the compensatory NAD^+^ production enzyme NAPRT, which we observed in half of our NB models, has been shown to mediate sensitivity in other cancer models and may be a contributing factor [9, 41–47]. Interestingly, NAPRT expression in cancer has been linked to the tissue of origin [55, 56], and other neuroendocrine tumors, which are derived from neural crest cells that also give rise to NB, have been shown to lack NAPRT expression [57] and exhibit high sensitivity to NAMPTis [58, 59]. Although we saw in vitro and in vivo responses to NB cells both with and without NAPRT expression, cell lines lacking NAPRT were more sensitive in vitro suggesting that NAPRT loss may confer greater sensitivity to NAMPTis in NB. Notably, the downstream effects of in vitro NAMPT inhibition were relatively consistent even across cell lines of differing sensitivities when treated with doses impairing proliferation. Interestingly, we did not observe a difference in sensitivity observed between our NAPRT expressing and non-expressing models in vivo. One possible explanation for this is that the dose we used in vivo exceeded the activity threshold for both models. Without human pharmacokinetic data on OT-82, the potential clinical significance of this remains unknown. However, future clinical investigation of NAMPTis in NB should include correlative biology studies to better understand the potential relationship between NAPRT status and NAMPTi response in this malignancy.

Alternatively, the downstream cell-type specific demands for NAD^+^ may contribute to NB dependency on NAMPT. Multiple reports, reviewed in [60], have demonstrated the deleterious effects of metabolic disruption on NB cells, including glycolytic inhibition [61], and simultaneous interference with glycolysis and mitochondrial metabolism [62]. We observed profound effects of NAMPT inhibition on the levels of glycolytic intermediates demonstrating that cytosolic NAD^+^ depletion alters glycolytic flux in both *MYCN* amplified and non-amplified NB models. Levels of both F6P and FBP were increased in all NB cell lines tested, whereas levels of 1,3-BPG were decreased in three of four NB cell lines following NAMPTi treatment, consistent with decreased GAPDH enzymatic activity [37, 63] likely resulting directly from depletion of cytosolic NAD^+^. Our analysis of cellular respiration revealed that mitochondrial oxygen consumption was also reduced in a majority of NB cell lines, suggesting that mitochondrial metabolism was disrupted by NAMPT inhibition. In addition, levels of several TCA cycle intermediates were altered, with decreases in α-ketoglutarate and succinate observed in most cell lines. Notably, both isocitrate dehydrogenase and the α-ketoglutarate dehydrogenase complex require NAD^+^ as an electron accepting cofactor. Concomitantly fumarate and malate were increased in all four cell lines, which may be a result of diminished NAD^+^ levels preventing the oxidation of malate to oxaloacetate by malate dehydrogenase. Decreased succinate levels are consistent with reduced entry of electrons into the electron transport chain (ETC) at the level of respiratory Complex II/succinate dehydrogenase, whereas diminished cellular NADH levels are consistent with decreased entry of electrons to the ETC via respiratory Complex I/NADH dehydrogenase. Together, our data identify a profound deleterious impact on cellular energy homeostasis caused by NAMPTi-mediated depletion of NAD^+^ and support impairment of glucose metabolism as a central component of NAMPTi response in NB.

Loss of poly ADP-ribosylation of proteins along with the disappearance of free cellular ADP-ribose that we observed following treatment with OT-82 represents another potential specific vulnerability in NB. Free ADP-ribose and cyclic ADP-ribose act as gating molecules for calcium (Ca^2+^) influx at the plasma membrane of cells via the transient receptor potential melastatin-related 2 (TRPM2) channel [64, 65] and their depletion would be expected to diminish cellular Ca^2+^ influx. Prior studies have demonstrated that depletion or functional disruption of TRPM2 in NB cells negatively impacts their growth in xenograft models [66, 67] indicating another potential vulnerability critical to NB cells.

Whether related to NAPRT expression, vulnerability to disruptions in energy metabolism, loss of poly ADP-ribosylation or other causes, the profound sensitivity of NB to NAMPT inhibition suggests that the therapeutic window for NAMPTis may be widened for patients with NB, and that they may experience benefit at substantially lower doses of NAMPTis than patients with other types of cancer. A comparison of our results with other preclinical studies of OT-82 revealed that NB models exhibit sensitivity equivalent to that of leukemia models, the disease for which OT-82 was initially developed [68, 69]. As no prior clinical trial testing of NAMPTis has included any patients with NB [12–18], our data provide a strong and actionable rationale for inclusion of NB patients in NAMPTi trials. Finally, our in vivo results also suggest that NAMPTi monotherapy on an intermittent dosing schedule may not be sufficiently active to cure patients with NB. Future studies to optimize the dosing regimen to provide either more continuous or a longer duration of NAMPT inhibition and/or to determine effective combination regimens will be critical to the future development of this class of agents for NB.

## Conclusions

In summary, these studies support NB as a therapeutically actionable indication for NAMPT inhibition. By integrating large-scale screening, mechanistic characterization, and orthotopic in vivo validation, this study establishes NAMPT-dependent NAD^+^ biosynthesis as a targetable vulnerability in neuroblastoma. The combination of broad sensitivity across molecularly diverse models, on-target metabolic disruption, and in vivo tumor regressions supports further evaluation of NAMPT inhibitors, including optimization of dosing strategies and rational combination approaches, for children with high-risk NB.

## Supplementary Information

Supplementary Material 1.

Supplementary Material 2.

Supplementary Material 3.

Supplementary Material 4.

Supplementary Material 5.

Supplementary Material 6.

Supplementary Material 7.

Supplementary Material 8: Supplemental Table S1.

Supplementary Material 9: Supplemental Table S2.

Supplementary Material 10: Supplemental Table S3.

Supplementary Material 11: Supplemental Dataset S1.

Supplementary Material 12: Supplemental Dataset S2.

Supplementary Material 13: Supplemental Dataset S3.

Supplementary Material 14: Supplemental Materials and Methods.

## Data Availability

The datasets supporting the conclusions of this article are available in the NCBI’s Gene Expression Omnibus (GEO) database under accession number GSE319723 (RNAseq data). Other data generated in this study are available within the article and supplementary data files. Additional data are available upon request from the corresponding author.

## Abbreviations

NB: Neuroblastoma
NAD^+^: Nicotinamide adenine dinucleotide
NAMPT: Nicotinamide phosphoribosyltransferase
ATP: Adenosine triphosphate
QPRT: Quinolinate phosphoribosyltransferase
NA: Nicotinic acid
NAPRT: Nicotinate phosphoribosyltransferase
NAM: Nicotinamide
NMN: Nicotinamide mononucleotide
NAMPTi: NAMPT inhibitor
EwS: Ewing sarcoma
RMS: Rhabdomyosarcoma
MIPE: Mechanism Interrogation Plate
PDX: Patient-derived xenograft
HPBCD: (2)-Hydroxypropyl-B-cyclodextrin
BCA: Bicinchoninic acid
ECAR: Extracellular acidification rate
OCR: Oxygen consumption rate
U/S: Ultrasound
BLI: Bioluminescence imaging
OXPHOS: Oxidative phosphorylation
GAPDH: Glyceraldehyde-3-phosphate dehydrogenase
F6P: Fructose-6-phosphate
FBP: Fructose-1,6-bisphosphate
1,3-BPG: 1,3-Bisphosphoglycerate
PARP: Poly (ADP-ribose) polymerase
SIRT1: Sirtuin 1
PI: Propidium iodide
ETC: Electron transport chain
Ca^2+^: Calcium
TRPM2: Transient receptor potential melastatin-related 2
