# Supplementary material for: Therapeutic NAMPT inhibition reveals a targetable metabolic vulnerability in neuroblastoma
Source: J Exp Clin Cancer Res. 2026 Aug 5;45:227. doi: 10.1186/s13046-026-03793-5 (PMC13726087; doi:10.1186/s13046-026-03793-5)
Supplement: Supplementary file 1 [file 13046_2026_3793_MOESM10_ESM.pdf]

Supplemental Table S3: NAMPT inhibitor IC_{50} values in neuroblastoma cell lines

Cell Line	OT-82 IC_{50} (Control = 100%)	KPT-9274 IC_{50} (Control = 100%)	Daporinad IC_{50} (Control = 100%)
SK-N-AS	2.5nM – 5nM	125nM – 250nM	2.5nM – 5nM
BE(2)C	1.6nM	250nM	1.6nM – 3.2nM
SY5Y	0.4nM – 0.8nM	20nM	0.4nM - 0.8nM
Kelly	0.1nM – 0.2nM	10nM	0.2nM – 0.4nM
IMR5	0.4nM – 0.8nM	20nM-30nM	0.8nM – 1.6nM
IMR32	0.2nM – 0.4nM	4nM-10nM	0.4nM – 0.8nM
KCNR	0.4nM – 0.8nM	10nM-20nM	0.8nM – 1.6nM
LAN5	0.4nM – 0.8nM	10nM-20nM	0.8nM – 1.6nM
SJNBL012407	1.6nM – 3.2nM	500nM-1000nM	3.2nM – 6.4nM
