# Supplementary material for: Therapeutic NAMPT inhibition reveals a targetable metabolic vulnerability in neuroblastoma
Source: J Exp Clin Cancer Res. 2026 Aug 5;45:227. doi: 10.1186/s13046-026-03793-5 (PMC13726087; doi:10.1186/s13046-026-03793-5)
Supplement: Supplementary file 2 [file 13046_2026_3793_MOESM14_ESM.pdf]

Supplementary Materials and Methods

Methods for Supplemental Data (RNA sequencing, biochemical analyses, cell cycle analysis, ROS assay, and tissue staining) are described below:

Total RNA Sequencing and Analysis

RNA was extracted using AllPrep DNA/RNA Micro Kit (Qiagen) according to manufacturer's instructions and DNase digestion was carried out using the RNase-Free DNase Set (Qiagen). Quality and quantity of RNA was assessed by Nanodrop and Agilent 4150 and 4200 TapeStation Systems at the Center for Cancer Research (CCR) Genomics Core. All samples were confirmed to have an integrity number greater than 8. Purified RNA samples were submitted to the Sequencing Facility at the Frederick National Laboratory for Cancer Research for total RNA-seq sequencing. RNA-Seq library was prepared using Illumina® Stranded Total RNA Prep, Ligation with Ribo-Zero Plus kit, followed by paired-end sequencing on the Illumina NovaSeq Xplus 10B platform with read length of 151bp. RNASeq data was processed using RENEE RNA-seq analysis pipeline (https://github.com/CCBR/RENEE) [1]. Briefly, the RNA-seq raw reads were trimmed for adaptors and low-quality bases using Cutadapt1.18 [2] and aligned to Human reference genome (hg38) using STAR2.7.6a [3] which was run in a two-passes manner. RSEM1.3.3 [4] was used for quantification of gene expression. Read count normalization and differential gene expression analysis were done using DESeq2 (version 1.42.1) [5]. Genes with a log2FoldChange >2 or <-2 and FDR less than 0.05 were considered differentially expressed. The statistical results from DESeq2 were used to rank the genes in gene set enrichment analysis (GSEA) from clusterProfiler package (version 4.10.1) [6] in R, where a false discovery rate of less than 0.25 and NES score bigger than 1.5 was considered a significant enrichment. The visualization for the GSEA results were generated using ggplot2 package (version 3.5.0). Transcripts per million (TPM) were used for data visualization in heatmaps using pheatmap package (version 1.0.12) in R.

Biochemical Analyses

The YSI 2950D Biochemistry Analyzer (Xylem Inc.) was used to measure glucose in the media of cells treated with OT-82 or DMSO (control). Cells were plated in triplicate in 24-well plates at 100,000 cells/well. Media were collected after 72 hours of treatment and analyzed per manufacturer's instructions.

Cell Cycle Analysis

Cells were plated at $1x10^6$ (SK-N-AS, BE(2)C) or $2x10^6$ (SY5Y, Kelly)/10-cm plate and allowed adherence overnight. Cells were treated with OT-82 and harvested at 24h, 48h, and 72h. Cells were fixed with ethanol and stained with PI/Triton X-100 staining solution containing DNAse-free RNAse A in PBS as described in [Gibson, 2020 [7]]. Cells were analyzed using CytoFLEX S Flow Cytometer and analyzed using FlowJo V.10 cell cycle modeling.

ROS assay

NB cells were plated in 96 well plates at 5,000 (SK-N-AS, BE(2)C) or 10,000 cells/well (SY5Y, Kelly) and treated the following day. OT-82 and NAC were co-administered. Menadione was used as a positive control and added 2h before endpoint. H_2O_2 substrate was added 2h before endpoint. ROS was measured using the ROSGlo H_2O_2 assay (Promega) per manufacturer's instructions.

Tissue Staining

Tumor tissue was fixed in 10% neutral buffered formalin for 3 days before being transferred to 70% ethanol. Fixed tissues were processed using an automatic processor, embedded in paraffin, sectioned on a manual microtome, and mounted on charged slides. Slides were dried in an 80°C oven for one h and stained with hematoxylin and eosin (H&E) using a Sakura® Tissue-Tek® Prisma™ automated stainer with commercial reagents. Slides were coverslipped using a Sakura® Tissue-Tek™ Glass® automatic coverslipper and dried prior to

review. H&E slides were evaluated in a blinded fashion by a single pathologist (BK). Slides were scanned at 20× magnification using an Aperio AT2 brightfield slide scanner (Leica Biosystems). Digital image analysis was performed using HALO image analysis software (Indica Labs, v4.1) with a Random Forest algorithm.

REFERENCES

1. Sevilla, S., et al., *RENEE: Rna sEquencing aNalysis pipElinE*. 2024, Zenodo.
2. Martin, M., *Cutadapt removes adapter sequences from high-throughput sequencing reads.* 2011, 2011. **17**(1): p. 3.
3. Dobin, A., et al., *STAR: ultrafast universal RNA-seq aligner.* Bioinformatics, 2013. **29**(1): p. 15-21.
4. Li, B. and C.N. Dewey, *RSEM: accurate transcript quantification from RNA-Seq data with or without a reference genome.* BMC Bioinformatics, 2011. **12**: p. 323.
5. Love, M.I., W. Huber, and S. Anders, *Moderated estimation of fold change and dispersion for RNA-seq data with DESeq2.* Genome Biol, 2014. **15**(12): p. 550.
6. Wu, T., et al., *clusterProfiler 4.0: A universal enrichment tool for interpreting omics data.* Innovation (Camb), 2021. **2**(3): p. 100141.
7. Gibson, A.E., et al., *Inhibition of nicotinamide phosphoribosyltransferase (NAMPT) with OT-82 induces DNA damage, cell death, and suppression of tumor growth in preclinical models of Ewing sarcoma.* Oncogenesis, 2020. **9**(9): p. 80.
