# Supplementary material for: Therapeutic NAMPT inhibition reveals a targetable metabolic vulnerability in neuroblastoma
Source: J Exp Clin Cancer Res. 2026 Aug 5;45:227. doi: 10.1186/s13046-026-03793-5 (PMC13726087; doi:10.1186/s13046-026-03793-5)
Supplement: Supplementary file 3 [file 13046_2026_3793_MOESM1_ESM.pdf]

Supplemental Figure S1

A

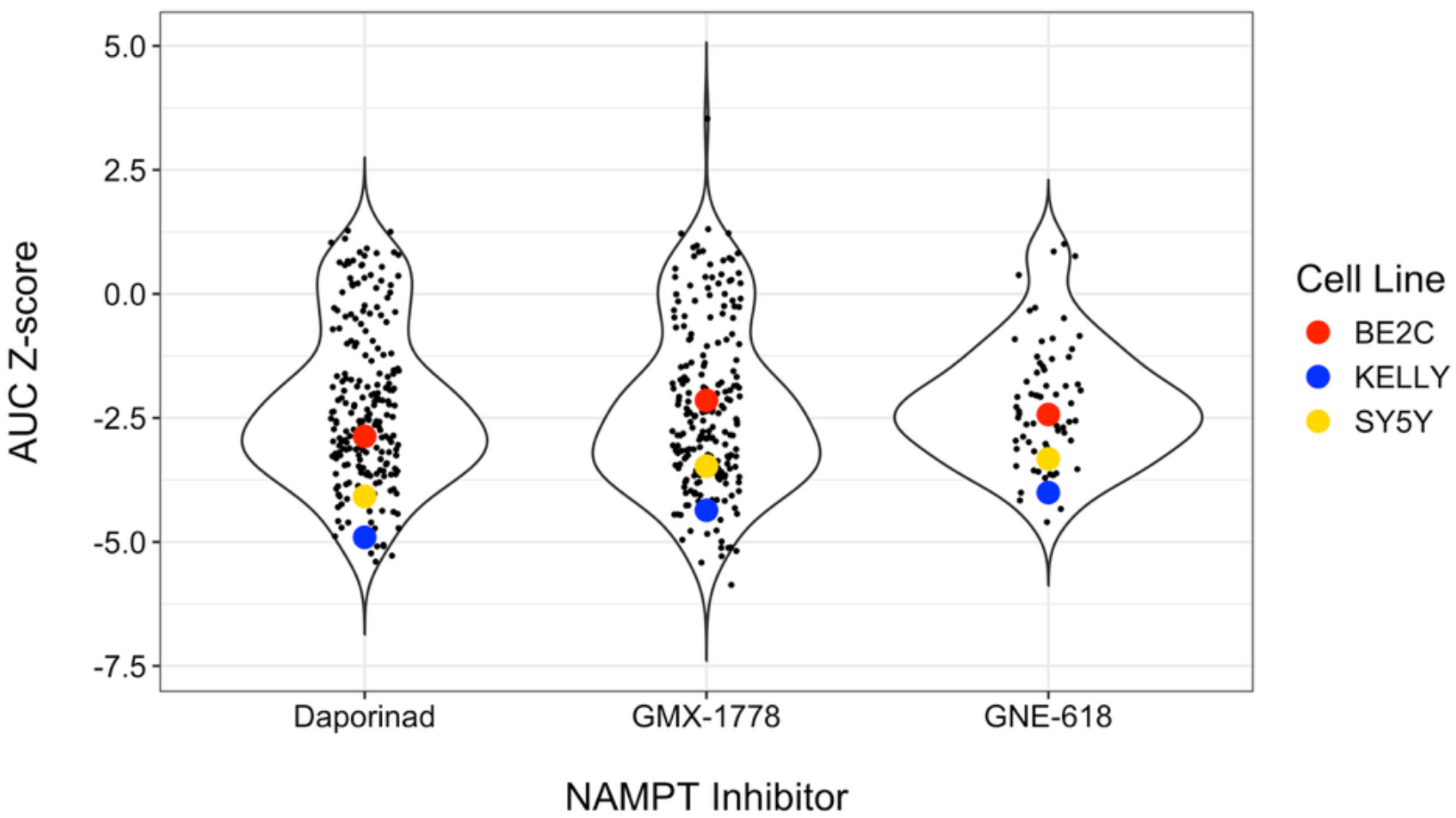

B

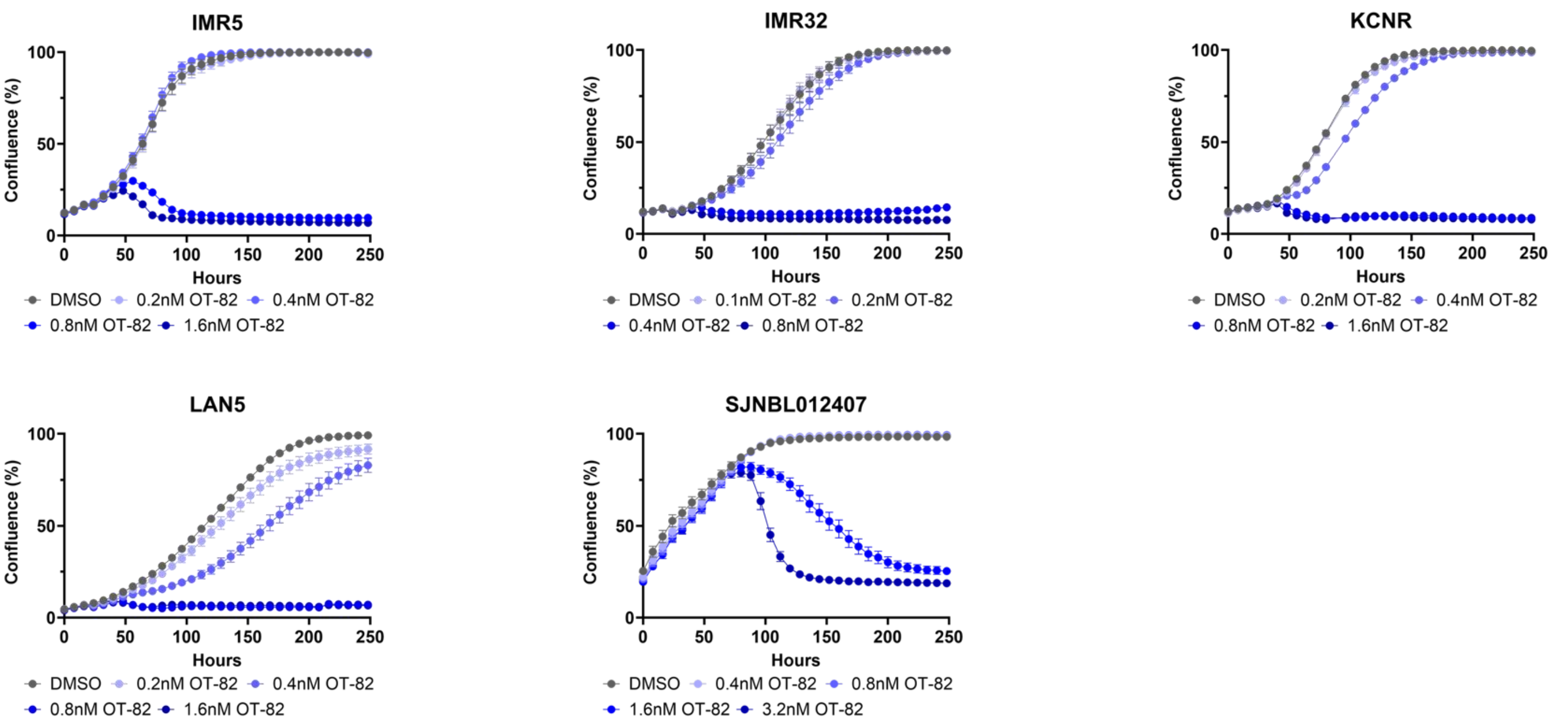

C

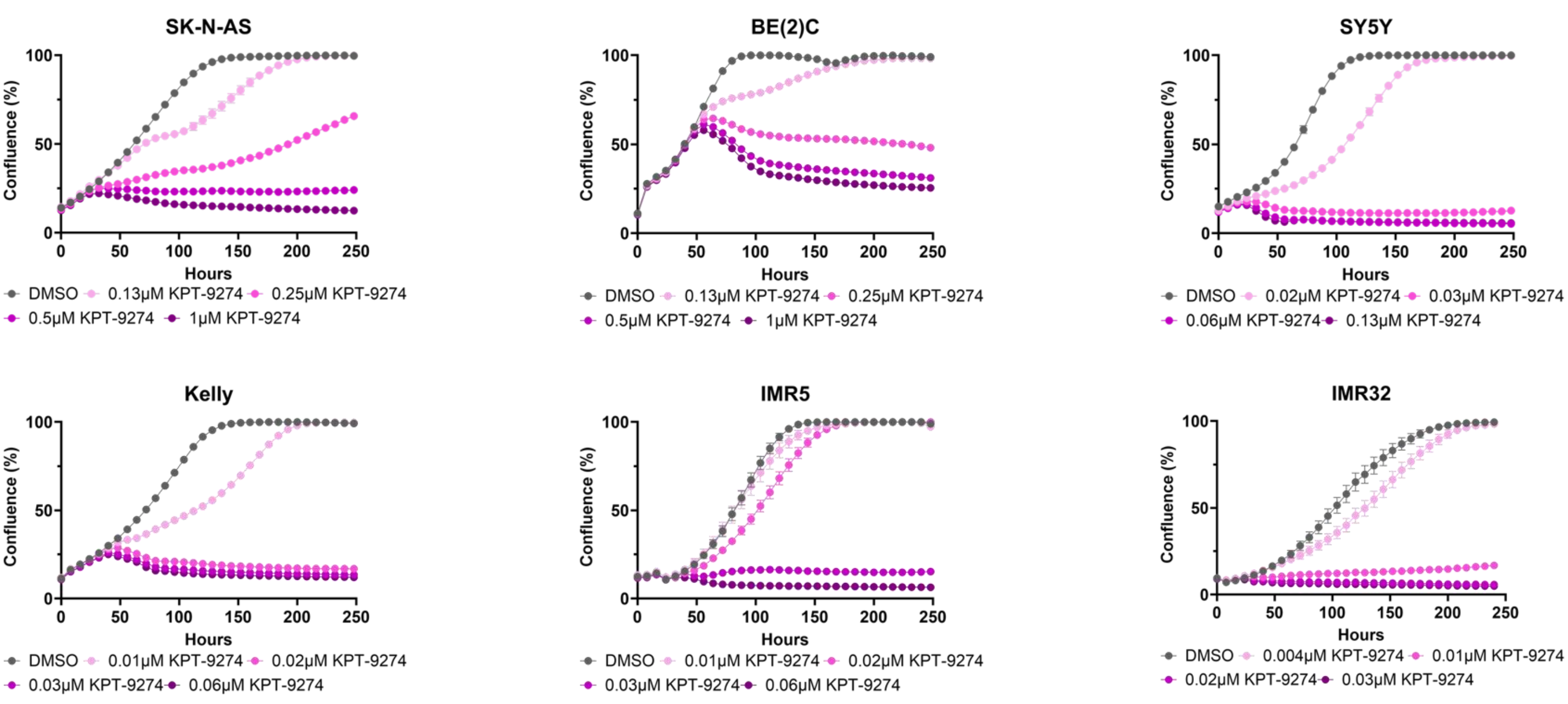

Supplemental Figure S1 (cont.)

C (cont.)

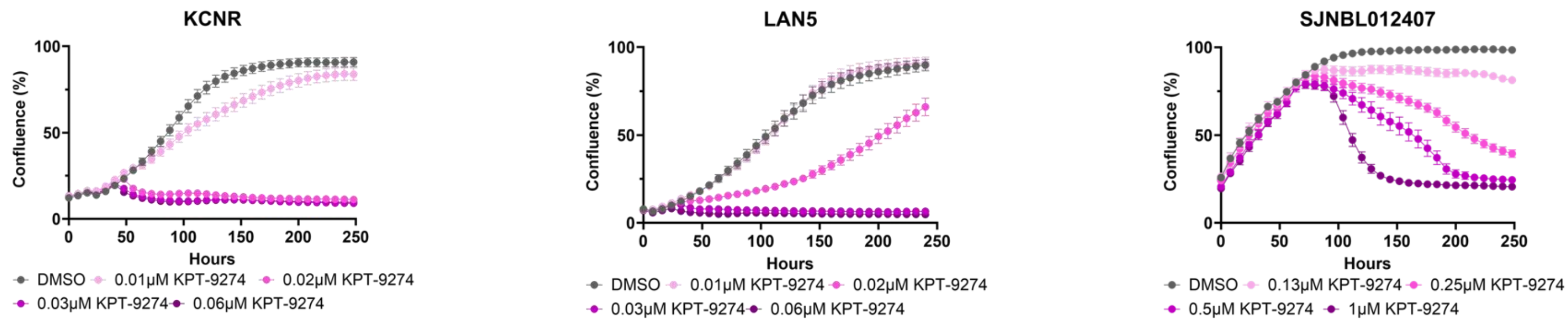

D

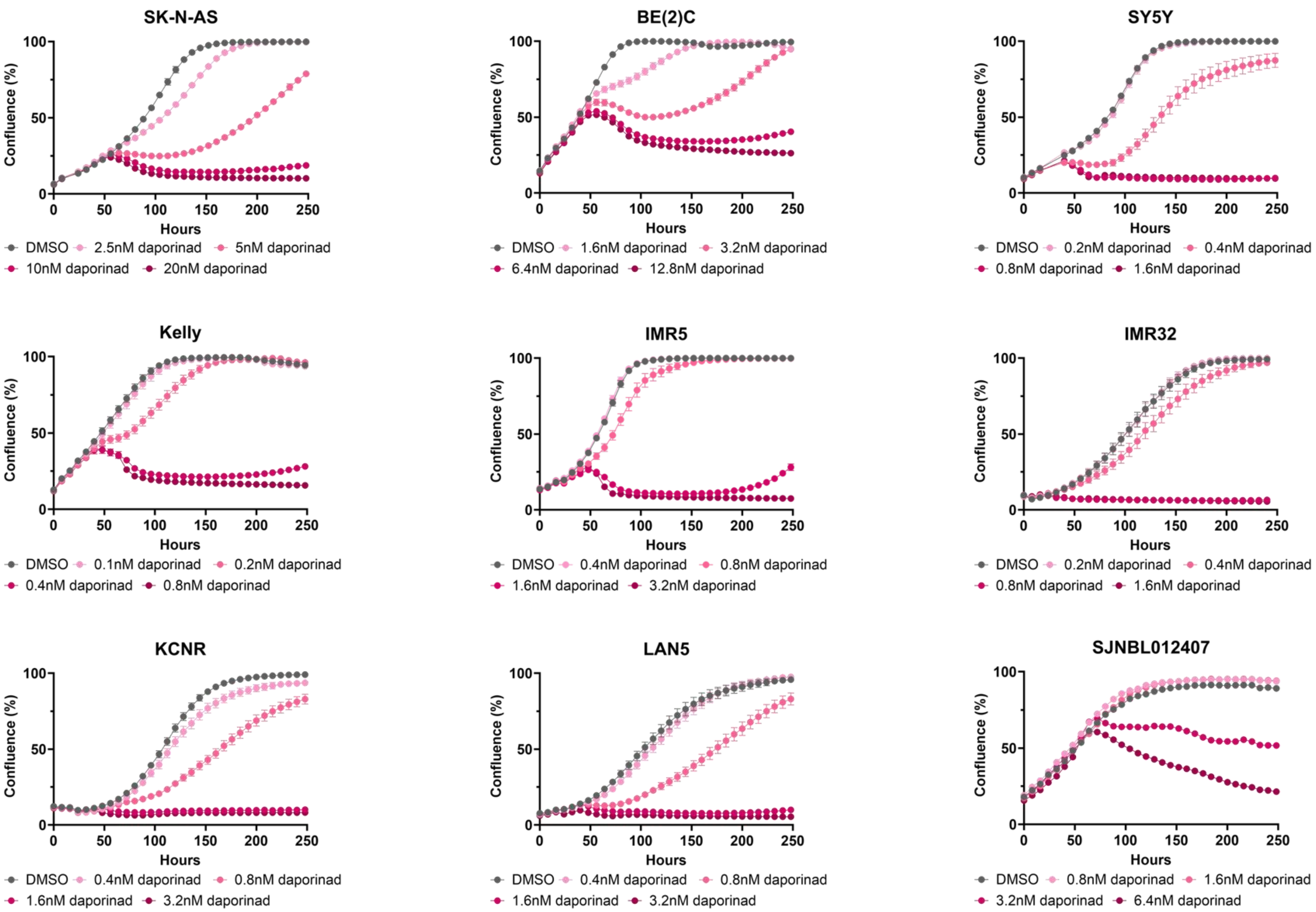

Supplemental Figure S1: (**A**) High-throughput drug screening data displayed onto 3 violin plots comparing the activity of three NAMPTis (daporinad, GMX-1778, and GNE-618) expressed as AUC Z-score. NB cell lines are highlighted in red, yellow, and blue. Daporinad and GMX-1778 datasets include n=200 cancer cell lines, and GNE-618 dataset include n=57 cancer cell lines. Each cell line is represented as a dot. (**B**) IncuCyte live cell proliferation analysis of NB cell lines IMR5 (10k/well), IMR32 (10k/well), KCNR (10k/well), LAN5 (10k/well), and SJNBL012407 (10k/well) treated with DMSO or varying doses of OT-82. Error bars represent SEM. Data shown is representative of one experiment. Each experiment was performed at least 3 times with at least 5 technical replicates. (**C**) IncuCyte live cell proliferation analysis of NB cell lines SK-N-AS (5k/well), BE(2)C (5k/well), SY5Y (10k/well), Kelly (10k/well), IMR5 (10k/well), IMR32 (10k/well), KCNR (10k/well), LAN5 (10k/well), and SJNBL012407 (10k/well) treated with DMSO or varying doses of KPT-9274. Error bars represent SEM. Data shown is representative of one experiment. Each experiment was performed at least 3 times with at least 5 technical replicates. (**D**) IncuCyte live cell proliferation analysis of NB cell lines SK-N-AS (5k/well), BE(2)C (5k/well), SY5Y (10k/well), Kelly (10k/well), IMR5 (10k/well), IMR32 (10k/well), KCNR (10k/well), LAN5 (10k/well), and SJNBL012407 (10k/well) treated with DMSO or varying doses of daporinad. Error bars represent SEM. Data shown is representative of one experiment. Each experiment was performed at least 3 times with at least 5 technical replicates.
