# Supplementary material for: Therapeutic NAMPT inhibition reveals a targetable metabolic vulnerability in neuroblastoma
Source: J Exp Clin Cancer Res. 2026 Aug 5;45:227. doi: 10.1186/s13046-026-03793-5 (PMC13726087; doi:10.1186/s13046-026-03793-5)
Supplement: Supplementary file 4 [file 13046_2026_3793_MOESM2_ESM.pdf]

Supplemental Figure S2

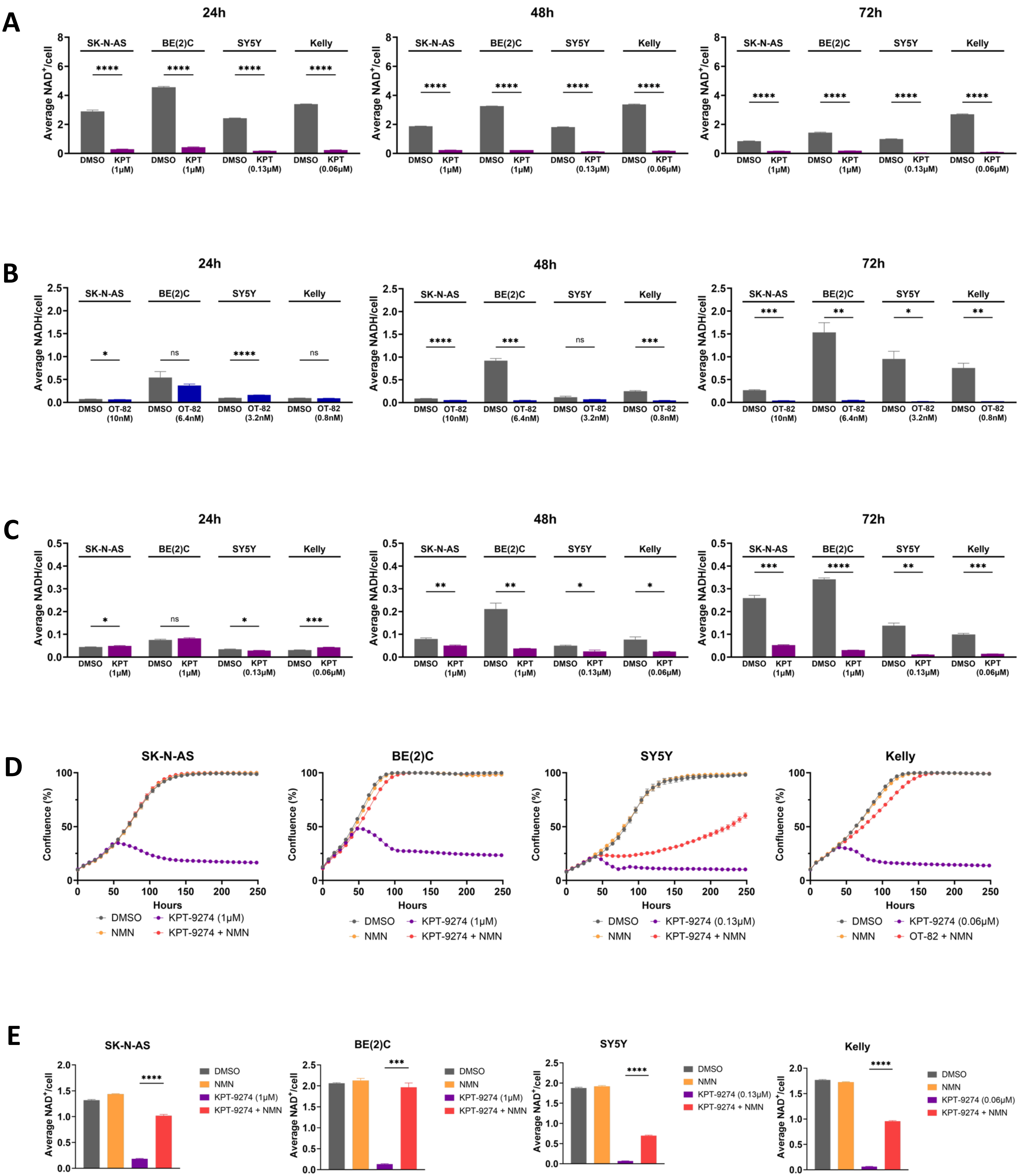

Supplemental Figure S2 (cont.)

F

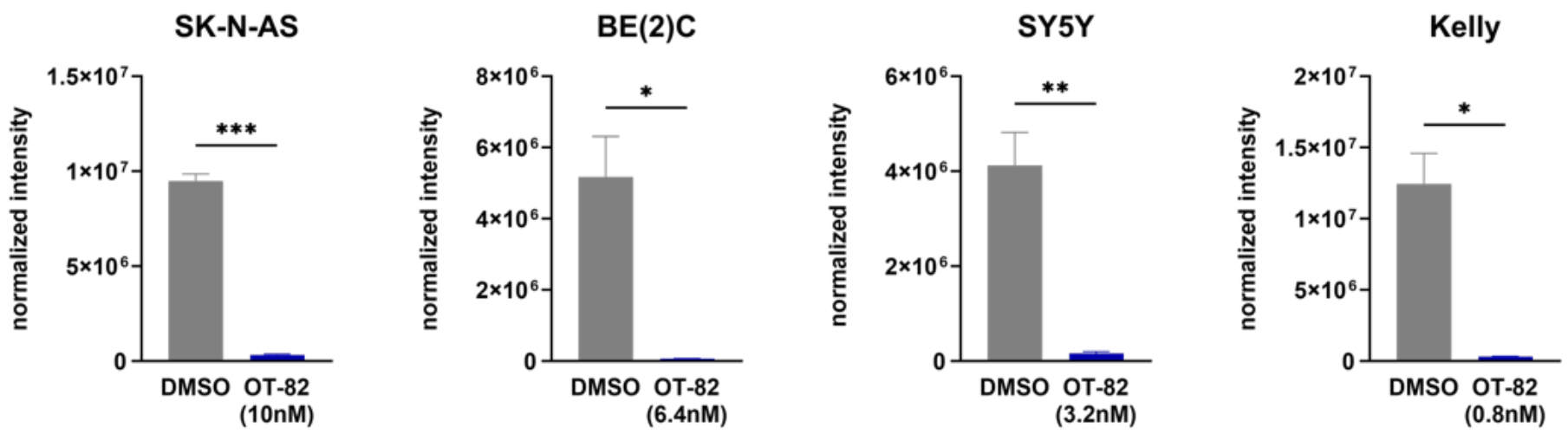

G

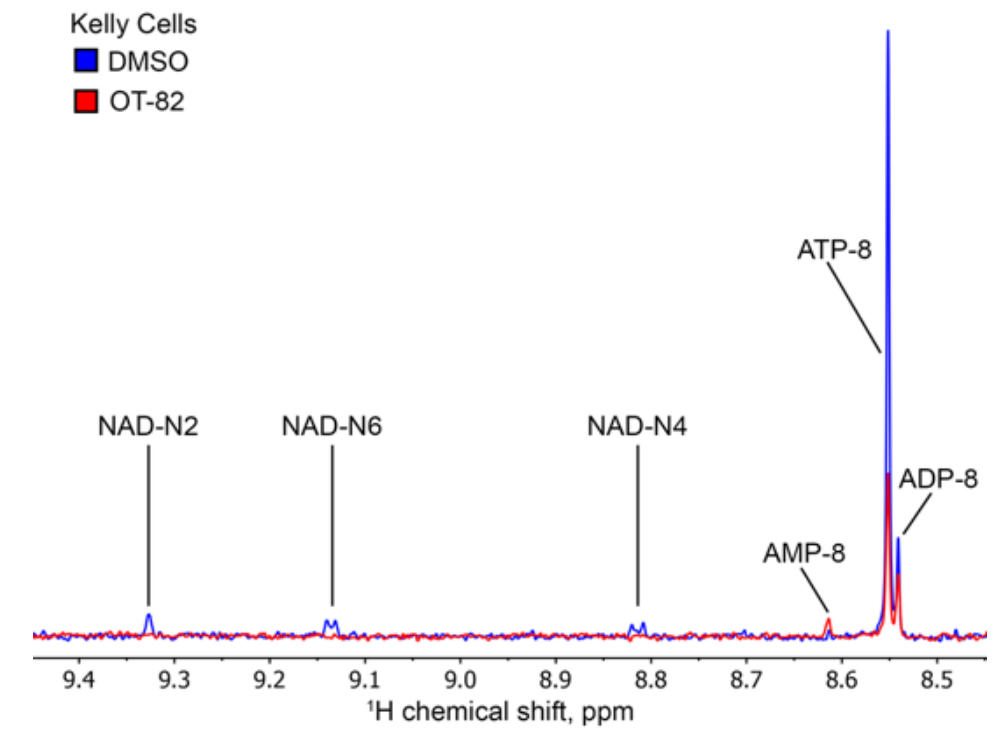

Supplemental Figure S2: (**A**) Average NAD⁺ level per cell in NB cell lines following treatment with DMSO (gray) or KPT-9274 (SK-N-AS 1μM, BE(2)C 1μM, SY5Y 0.13μM, and Kelly 0.06μM) (purple) at 24h, 48h, and 72h. Error bars represent SEM. Each experiment was performed at least 3 times with at least 4 technical replicates. (**B**) Average NADH level per cell number in NB models at 24h, 48h, and 72h after treatment with DMSO (gray) or OT-82 (SK-N-AS 10nM, BE(2)C 6.4nM, SY5Y 3.2nM, and Kelly 0.8nM) (blue). Error bars represent SEM. Each experiment was performed at least 3 times with at least 4 technical replicates. (**C**) Average NADH level per cell in NB cell lines following treatment with DMSO (gray) or KPT-9274 (SK-N-AS 1μM, BE(2)C 1μM, SY5Y 0.13μM, and Kelly 0.06μM) (purple) at 24h, 48h, and 72h. Error bars represent SEM. Each experiment was performed at least 3 times with at least 4 technical replicates. (**D**) IncuCyte live cell analysis measuring proliferation of NB cell lines treated with DMSO (gray), 1mM NMN (yellow), KPT-9274 (SK-N-AS 1μM, BE(2)C 1μM, SY5Y 0.13μM, and Kelly 0.06μM) (purple), or cotreatment with KPT-9274 and NMN (red). Error bars represent SEM. Data shown is representative of one experiment. Each experiment was performed at least 3 times with at least 5 technical replicates. (**E**) Average NAD⁺ level per cell in NB cell lines following treatment of DMSO (gray), 1mM NMN (yellow), KPT-9274 (SK-N-AS 1μM, BE(2)C 1μM, SY5Y 0.13μM, and Kelly 0.06μM) (purple), or cotreatment with KPT-2974 and NMN at 72h. Each experiment was performed at least 3 times with at least 4 technical replicates. (**F**) ICMS analysis of NADH levels in NB cell lines treated for 24h with DMSO or OT-82 (SK-N-AS 10nM, BE(2)C 6.4nM, SY5Y 3.2nM, and Kelly 0.8nM) expressed as normalized intensity. Each experiment had 3 technical replicates. Error bars represent SD. (**G**) Representative 1H NMR analysis of Kelly cells following 24h treatment with DMSO (blue) or OT-82 (red). The singlet resonance at 9.33ppm, and doublet resonances at 9.13ppm and 8.81ppm arise from the second, sixth and fourth positions of the nicotinamide moieties of NAD+. The singlet resonances at 8.61, 8.55, and 8.54 ppm arise from the ribose moiety of AMP, ATP, and ADP, respectively. Welch's t-test was performed for each experiment. ****$p < 0.0001$; ***$p < 0.001$; **$p < 0.01$; *$p < 0.05$.
