# Supplementary material for: Therapeutic NAMPT inhibition reveals a targetable metabolic vulnerability in neuroblastoma
Source: J Exp Clin Cancer Res. 2026 Aug 5;45:227. doi: 10.1186/s13046-026-03793-5 (PMC13726087; doi:10.1186/s13046-026-03793-5)
Supplement: Supplementary file 5 [file 13046_2026_3793_MOESM3_ESM.pdf]

Supplemental Figure S3

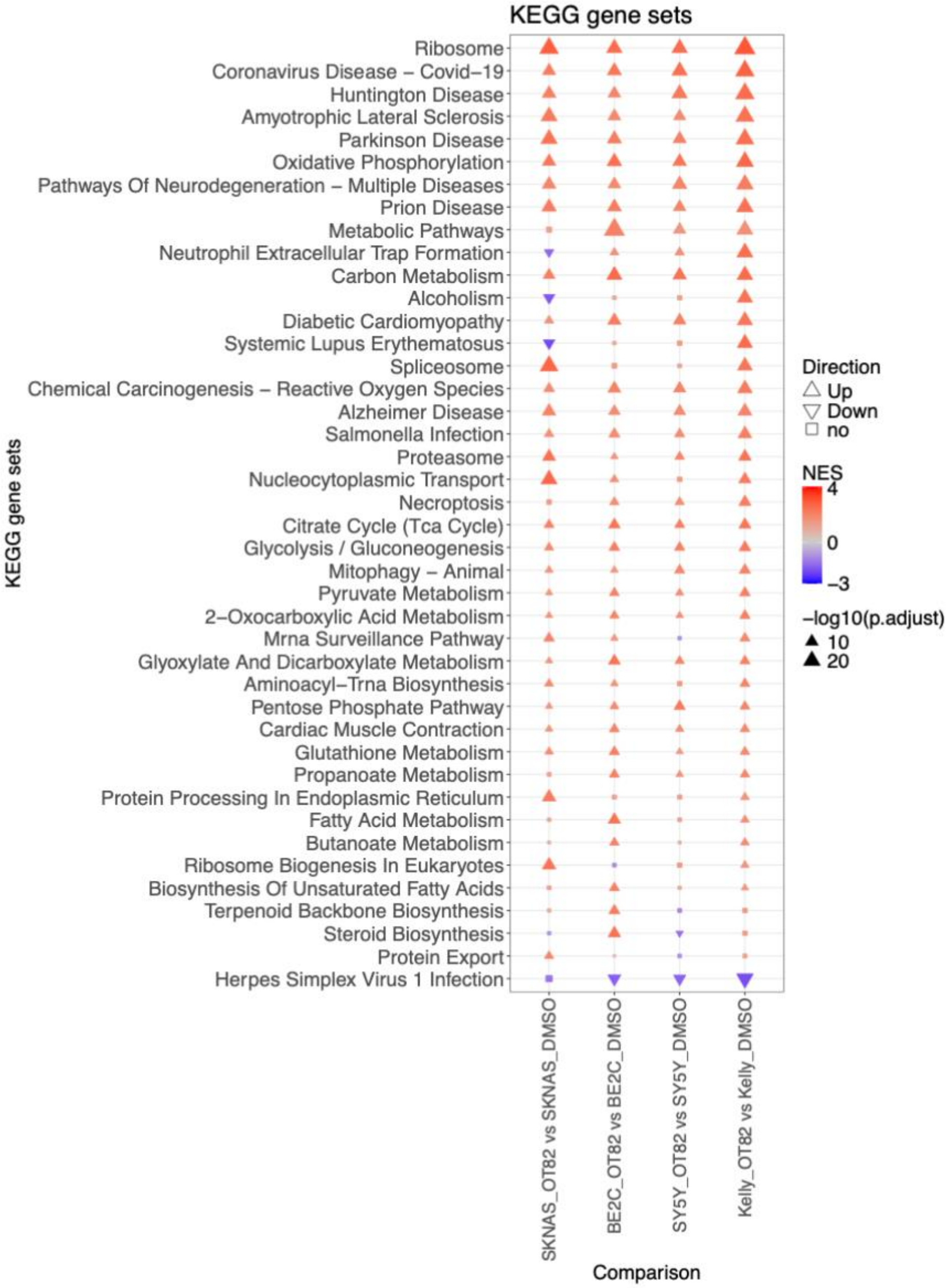

Supplemental Figure S3: Gene set enrichment analysis of NB cells show an upregulation of genes related to metabolic pathways. KEGG pathway analysis of NB cell lines treated with DMSO or OT-82 (SK-N-AS 10nM, BE(2)C 6.4nM, SY5Y 3.2nM, and Kelly 0.8nM) at 24h.
