# Supplementary material for: Therapeutic NAMPT inhibition reveals a targetable metabolic vulnerability in neuroblastoma
Source: J Exp Clin Cancer Res. 2026 Aug 5;45:227. doi: 10.1186/s13046-026-03793-5 (PMC13726087; doi:10.1186/s13046-026-03793-5)
Supplement: Supplementary file 6 [file 13046_2026_3793_MOESM4_ESM.pdf]

Supplemental Figure S4

A

	SK-N-AS			BE(2)C			SY5Y			Kelly		
	DMSO	OT-82	Oligo	DMSO	OT-82	Oligo	DMSO	OT-82	Oligo	DMSO	OT-82	Oligo
p-AMPK	1	2.541864	1.959135	1	1.952466	1.998432	1	1.600342	2.998487	1	1.444551	1.064587
p-raptor	1	1.752747	1.455939	1	1.840391	2.010425	1	1.72034	1.173987	1	1.603672	1.331529
t-AMPK	1	0.942311	0.601351	1	0.791866	0.605186	1	0.895458	0.88813	1	1.100111	1.215654
t-raptor	1	1.571718	1.435941	1	1.004847	0.832404	1	0.820271	0.959979	1	1.199509	1.797992

B

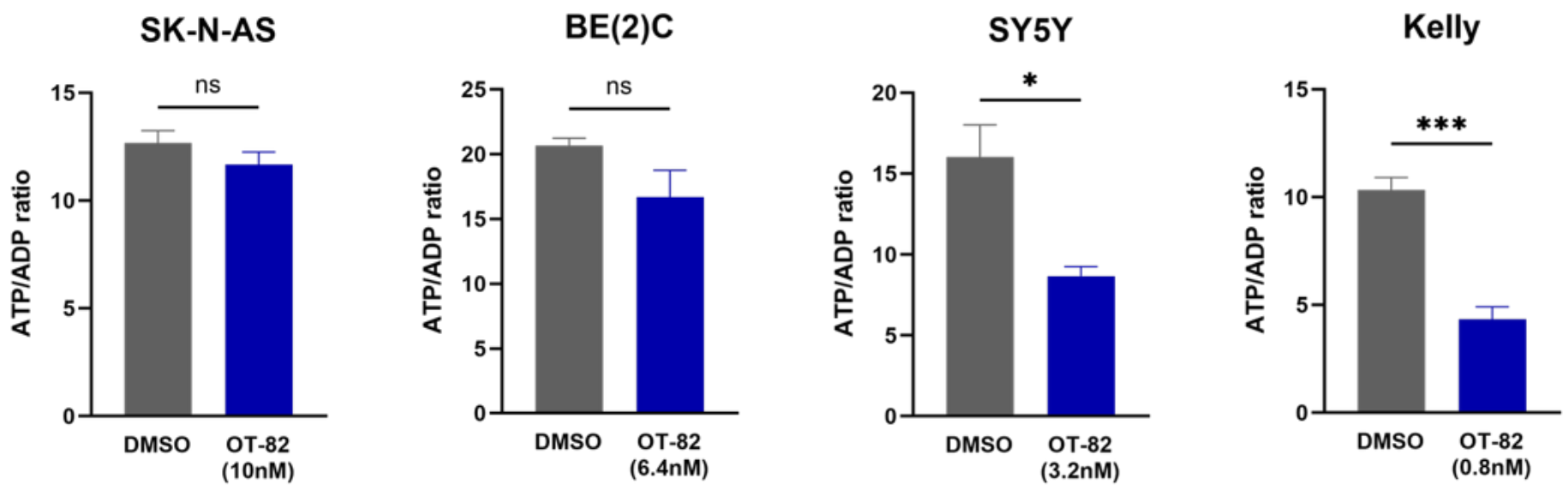

C

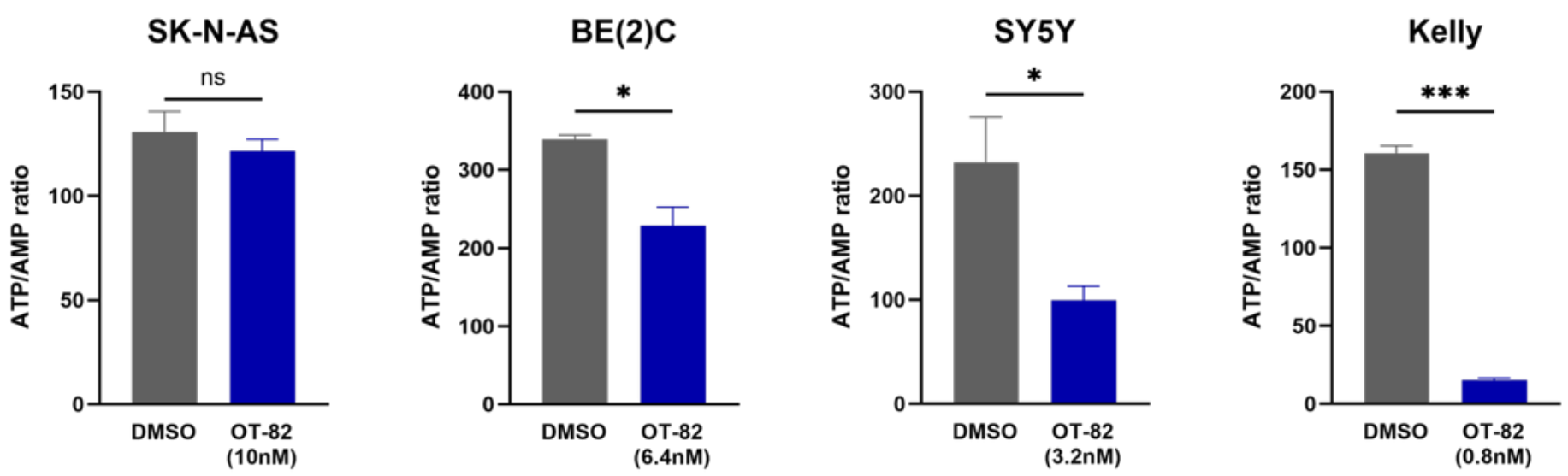

D

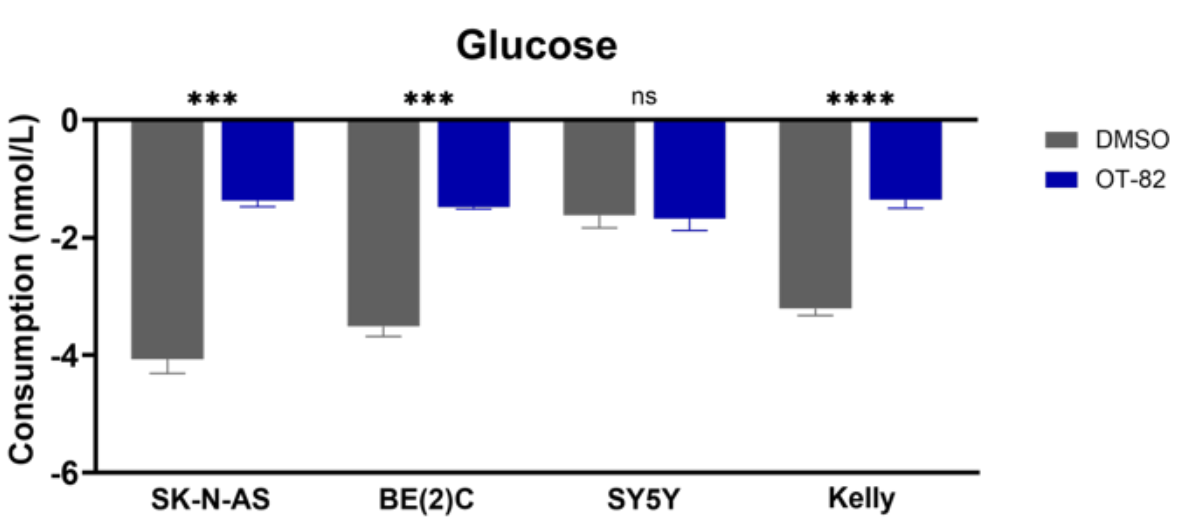

E

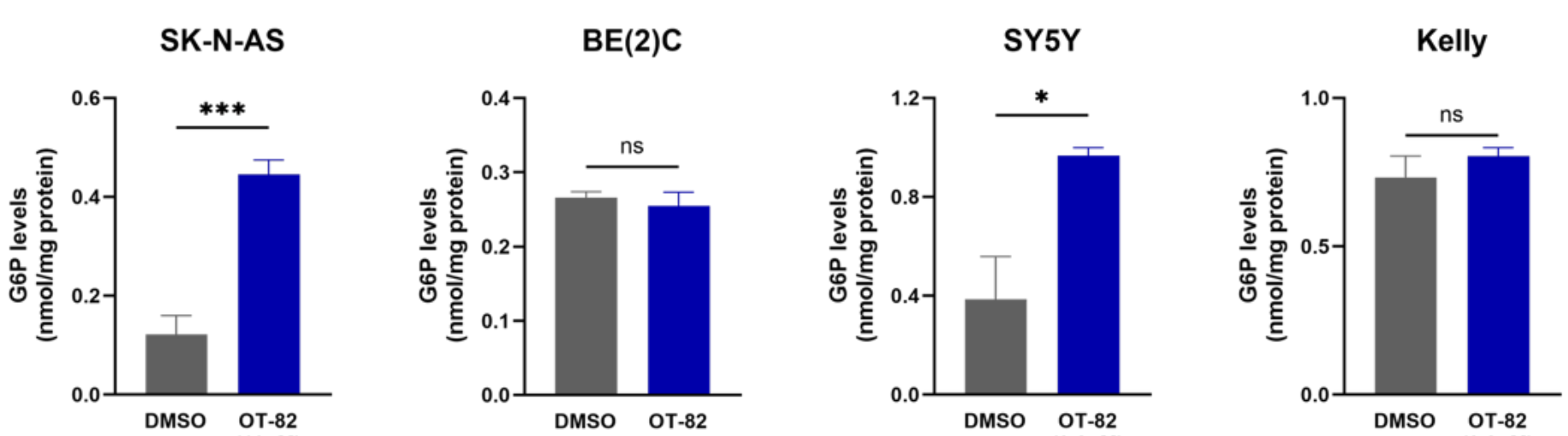

F

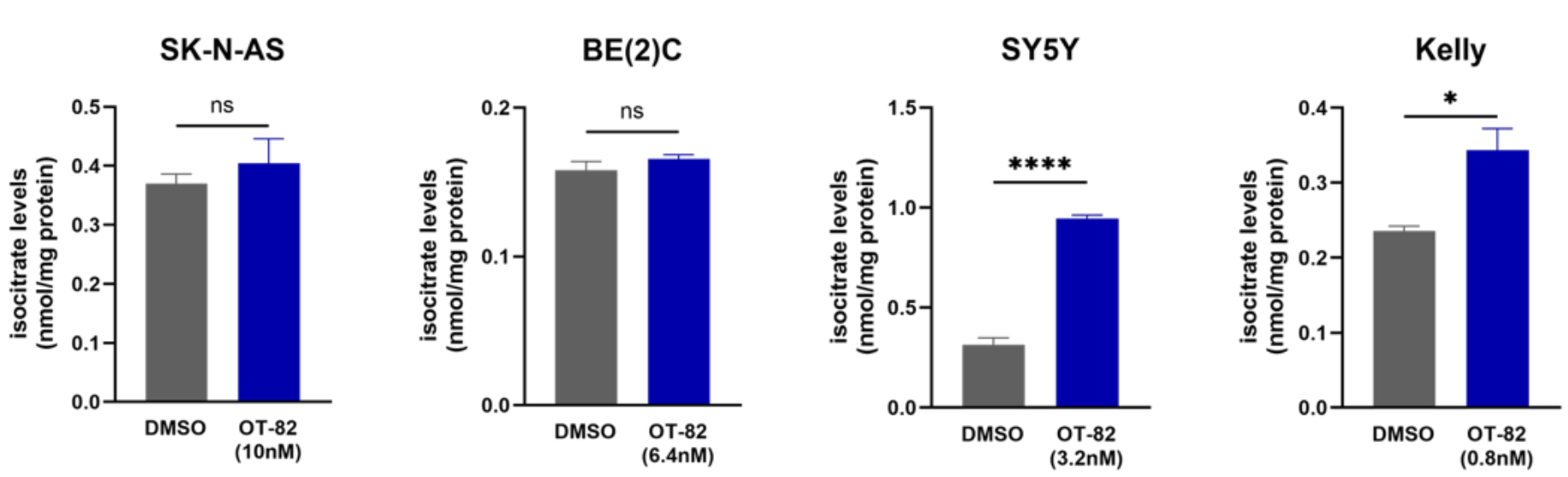

Supplemental Figure S4: (**A**) Quantification of immunoblot in Figure 2B showing pT172 AMPK,pSer792 raptor, total AMPK, and total raptor from one set of representative experiments in NB cells treated with DMSO, OT-82 (SK-N-AS 10nM, BE(2)C 6.4nM, SY5Y 3.2nM, and Kelly 0.8nM) (blue) for 24h, or positive control oligomycin 10 uM for 1hr. GAPDH is loading control. Each experiment was performed at least 2 times. (**B**) ATP/ADP ratios of NB cells treated with DMSO (gray) or OT-82 (SK-N-AS 10nM, BE(2)C 6.4nM, SY5Y 3.2nM, and Kelly 0.8nM) (blue) at 24h as measured by ICMS. Each experiment had 3 technical replicates. Error bars represent SD. (**C**) ATP/AMP ratios of NB cells treated with DMSO (gray) or OT-82 (SK-N-AS 10nM, BE(2)C 6.4nM, SY5Y 3.2nM, and Kelly 0.8nM) (blue) at 24h as measured by ICMS. Each experiment had 3 technical replicates. Error bars represent SD. (**D**) YSI analysis of glucose consumption in NB models treated with DMSO (gray) or OT-82 (SK-N-AS 10nM, BE(2)C 6.4nM, SY5Y 3.2nM, and Kelly 0.8nM) (blue) at 72h. Each experiment had 4 technical replicates. Error bars represent SD. (**E**) ICMS analysis of glucose-6-phosphate metabolite levels in NB models treated with DMSO (gray) or OT-82 (SK-N-AS 10nM, BE(2)C 6.4nM, SY5Y 3.2nM, and Kelly 0.8nM) (blue) at 24h. Each experiment had 3 technical replicates. Error bars represent SD. (**F)** ICMS analysis of isocitrate metabolite levels in NB models treated with DMSO (gray) or OT-82 (SK-N-AS 10nM, BE(2)C 6.4nM, SY5Y 3.2nM, and Kelly 0.8nM) (blue) at 24h. Each experiment had 3 technical replicates. Error bars represent SD. Welch's t-test was performed for each experiment. ****, $P < 0.0001$, ***, $P < 0.001$, *, $P < 0.05$.
