# Supplementary material for: Therapeutic NAMPT inhibition reveals a targetable metabolic vulnerability in neuroblastoma
Source: J Exp Clin Cancer Res. 2026 Aug 5;45:227. doi: 10.1186/s13046-026-03793-5 (PMC13726087; doi:10.1186/s13046-026-03793-5)
Supplement: Supplementary file 7 [file 13046_2026_3793_MOESM5_ESM.pdf]

Supplemental Figure S5

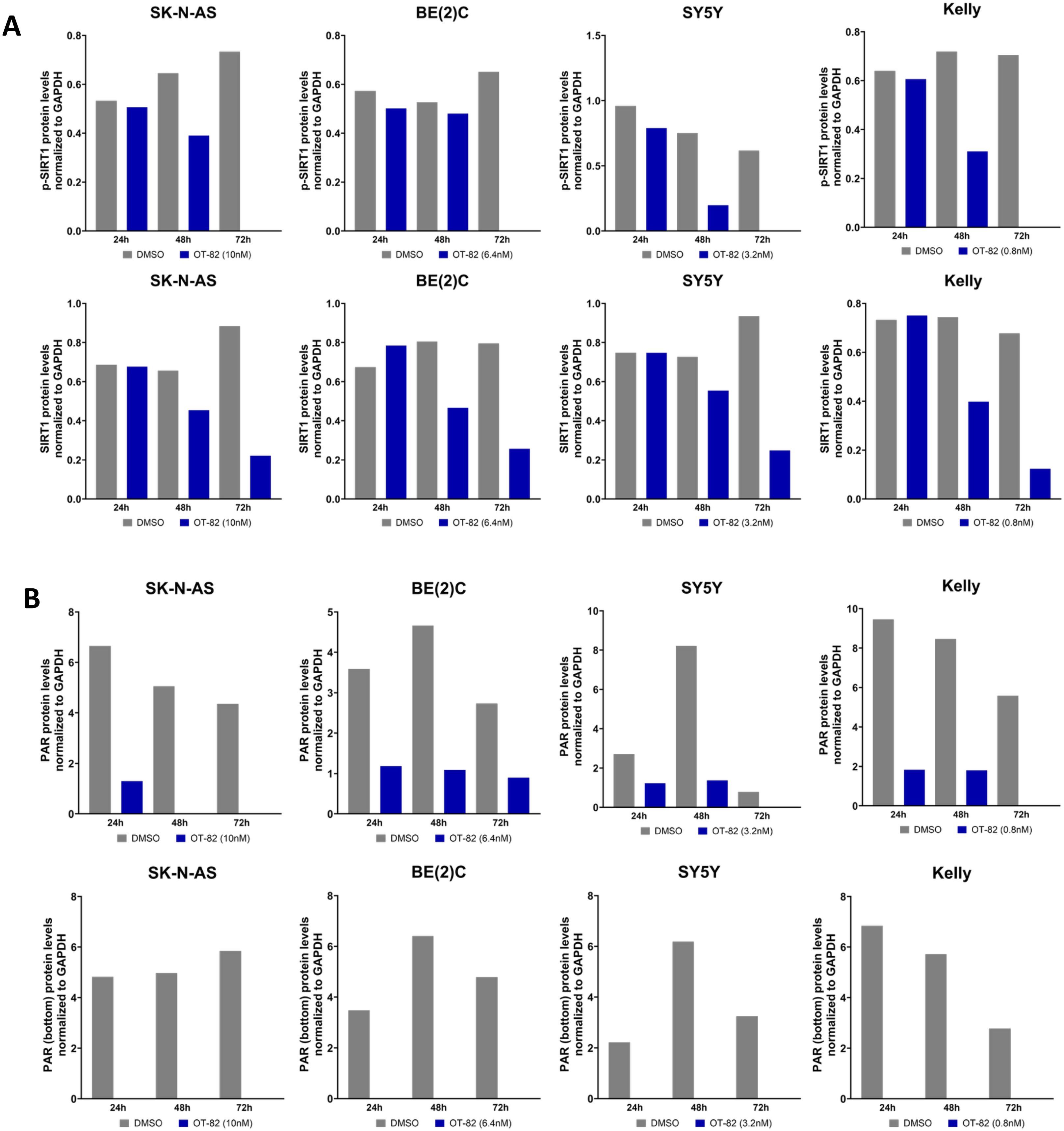

Supplemental Figure S5: (**A**) Quantification of p-SIRT1 protein levels normalized to loading control GAPDH from one set of representative experiments. Each experiment was performed at least 3 times. (**B**) Quantification of PAR protein levels normalized to loading control GAPDH from one set of representative experiments. Each experiment was performed at least 3 times.
