# Supplementary material for: Therapeutic NAMPT inhibition reveals a targetable metabolic vulnerability in neuroblastoma
Source: J Exp Clin Cancer Res. 2026 Aug 5;45:227. doi: 10.1186/s13046-026-03793-5 (PMC13726087; doi:10.1186/s13046-026-03793-5)
Supplement: Supplementary file 8 [file 13046_2026_3793_MOESM6_ESM.pdf]

Supplemental Figure S6

A

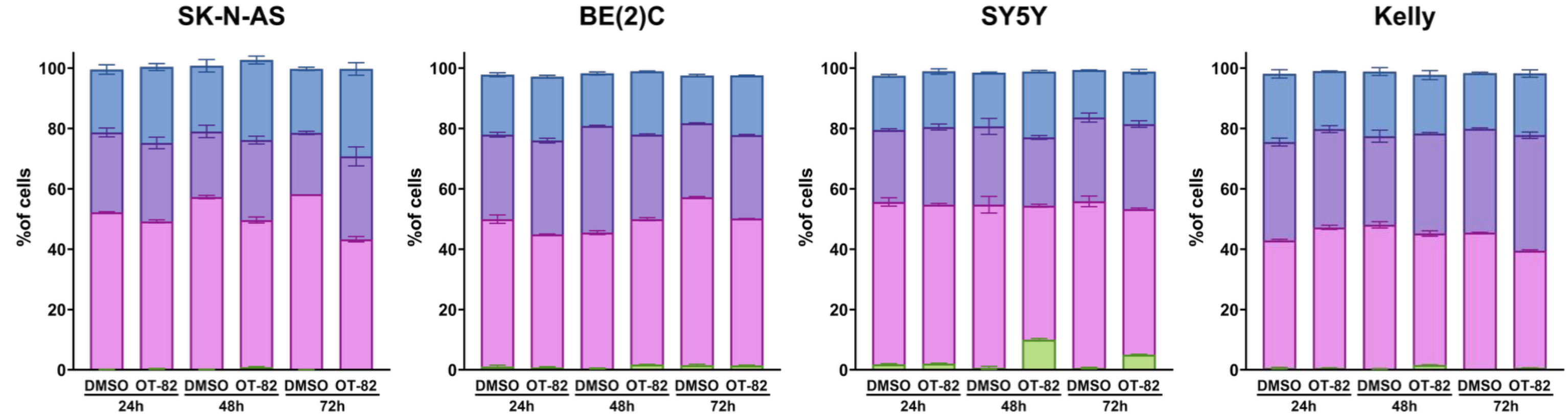

B

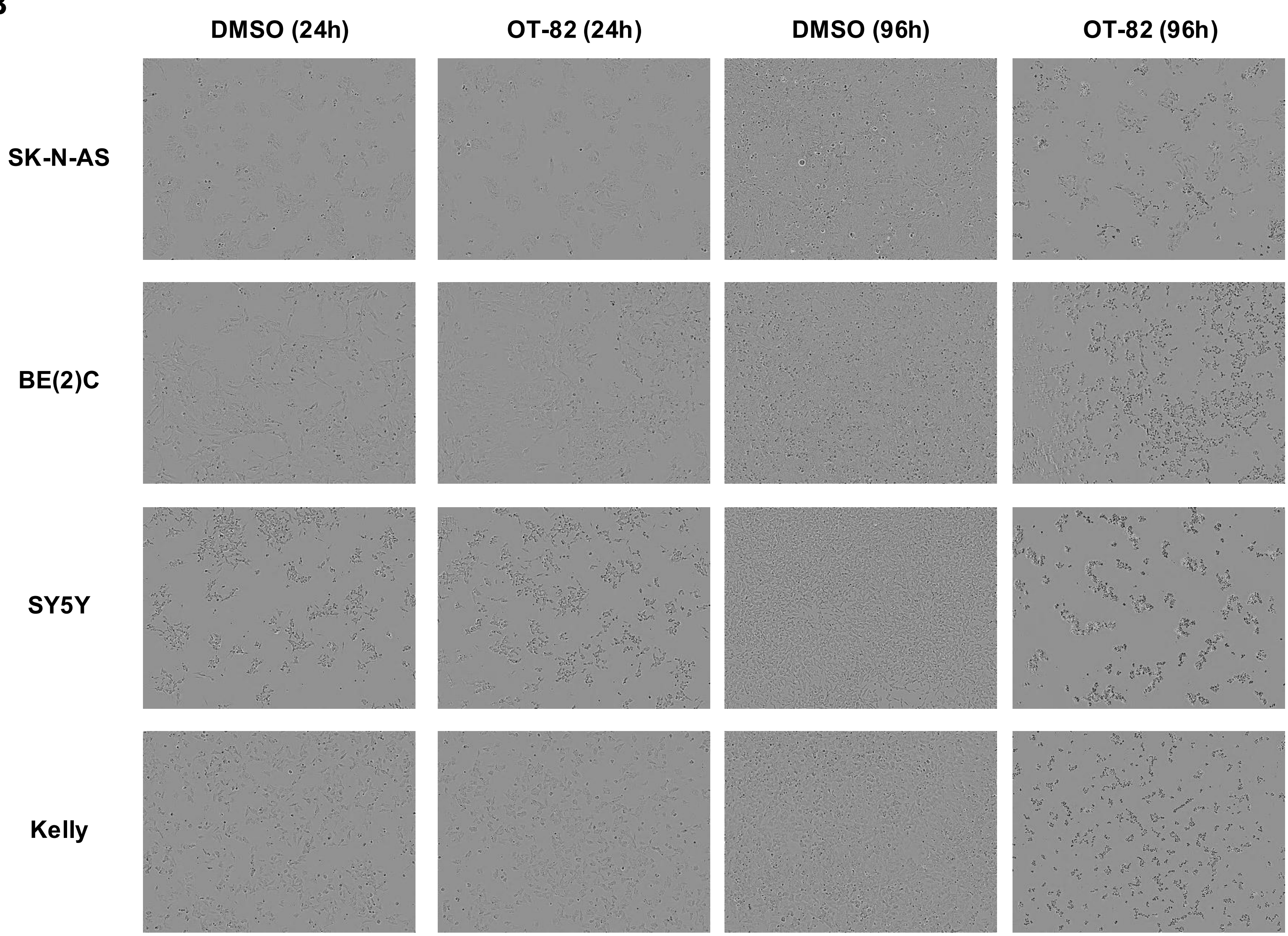

C

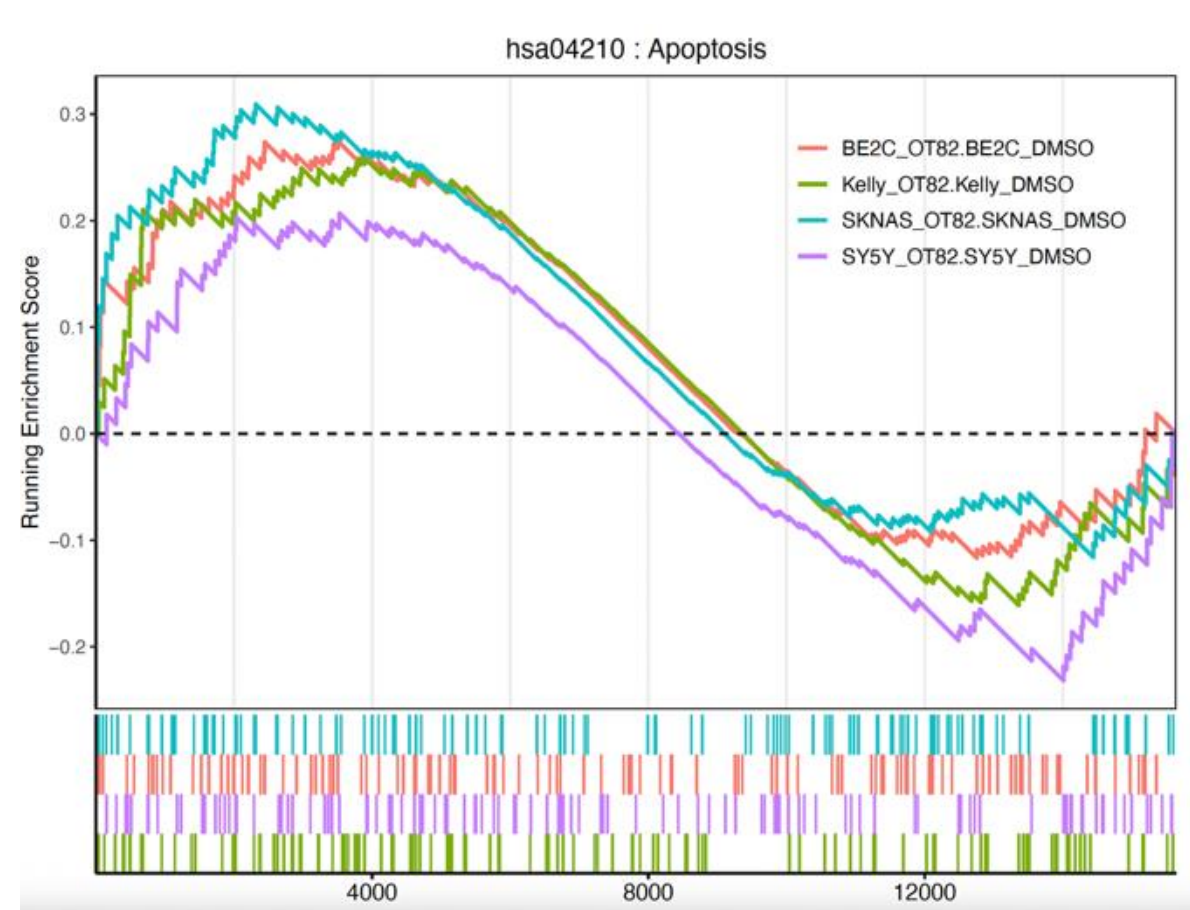

Supplemental Figure S6 (cont.)

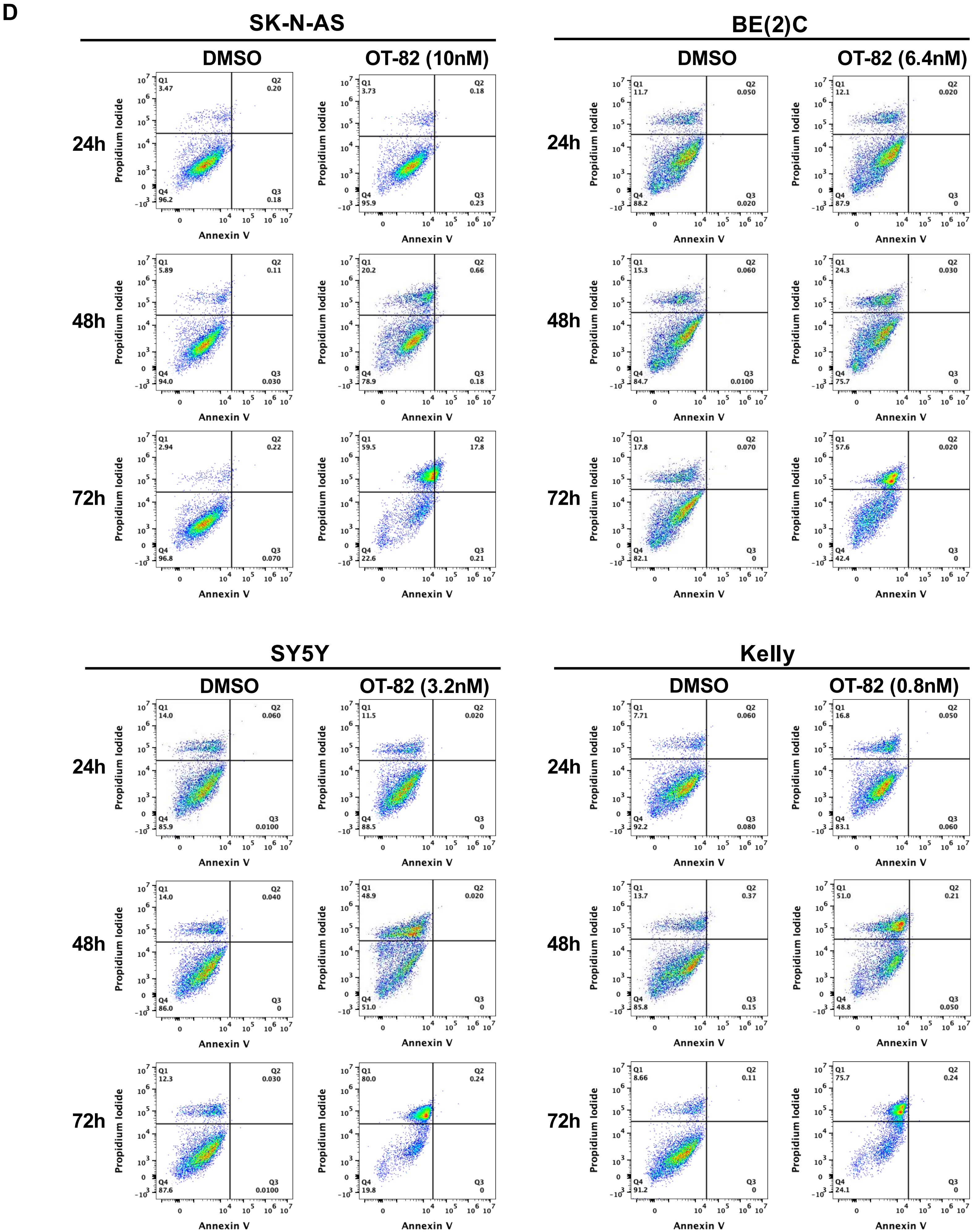

Supplemental Figure S6 (cont.)

E

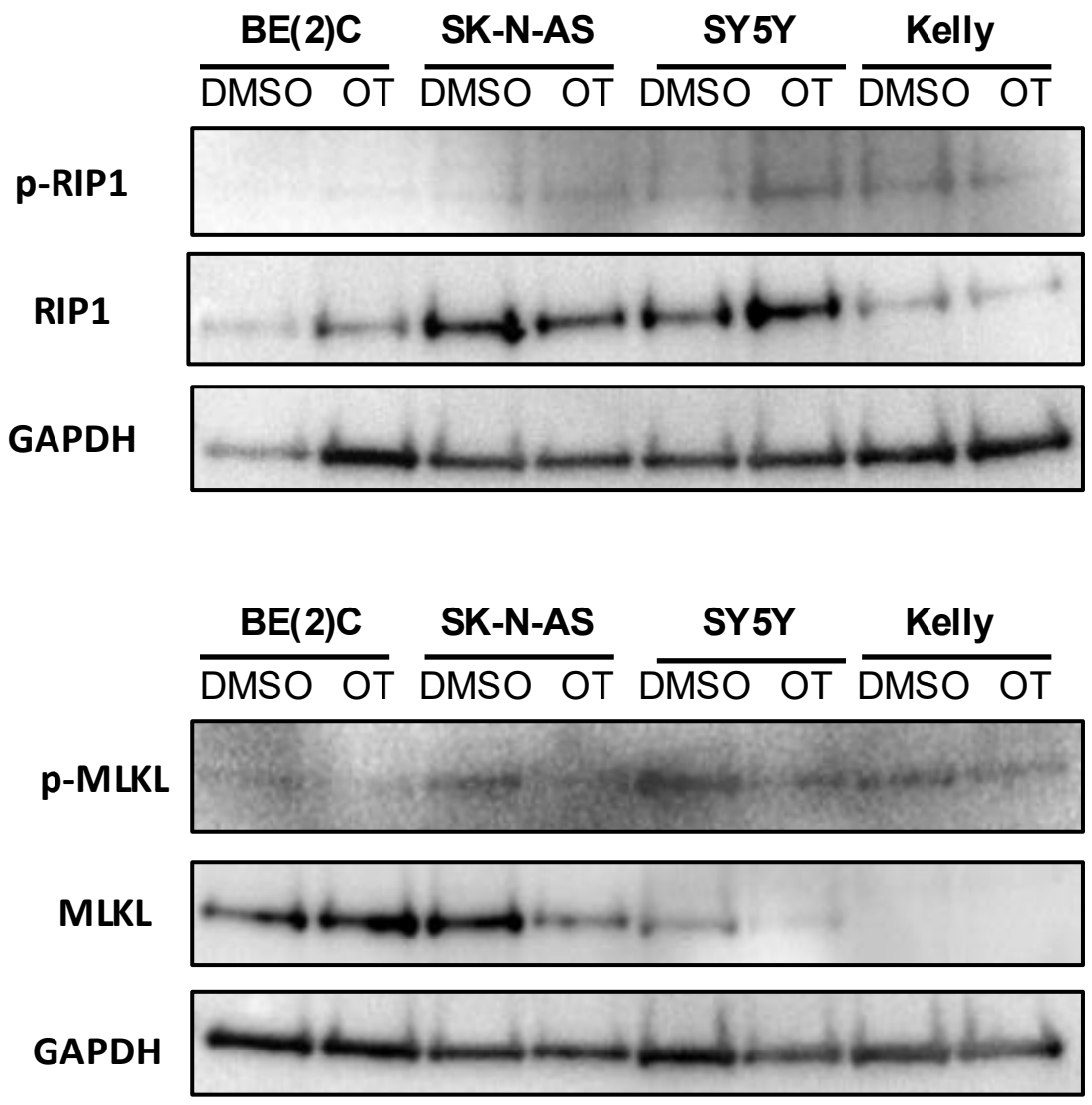

F

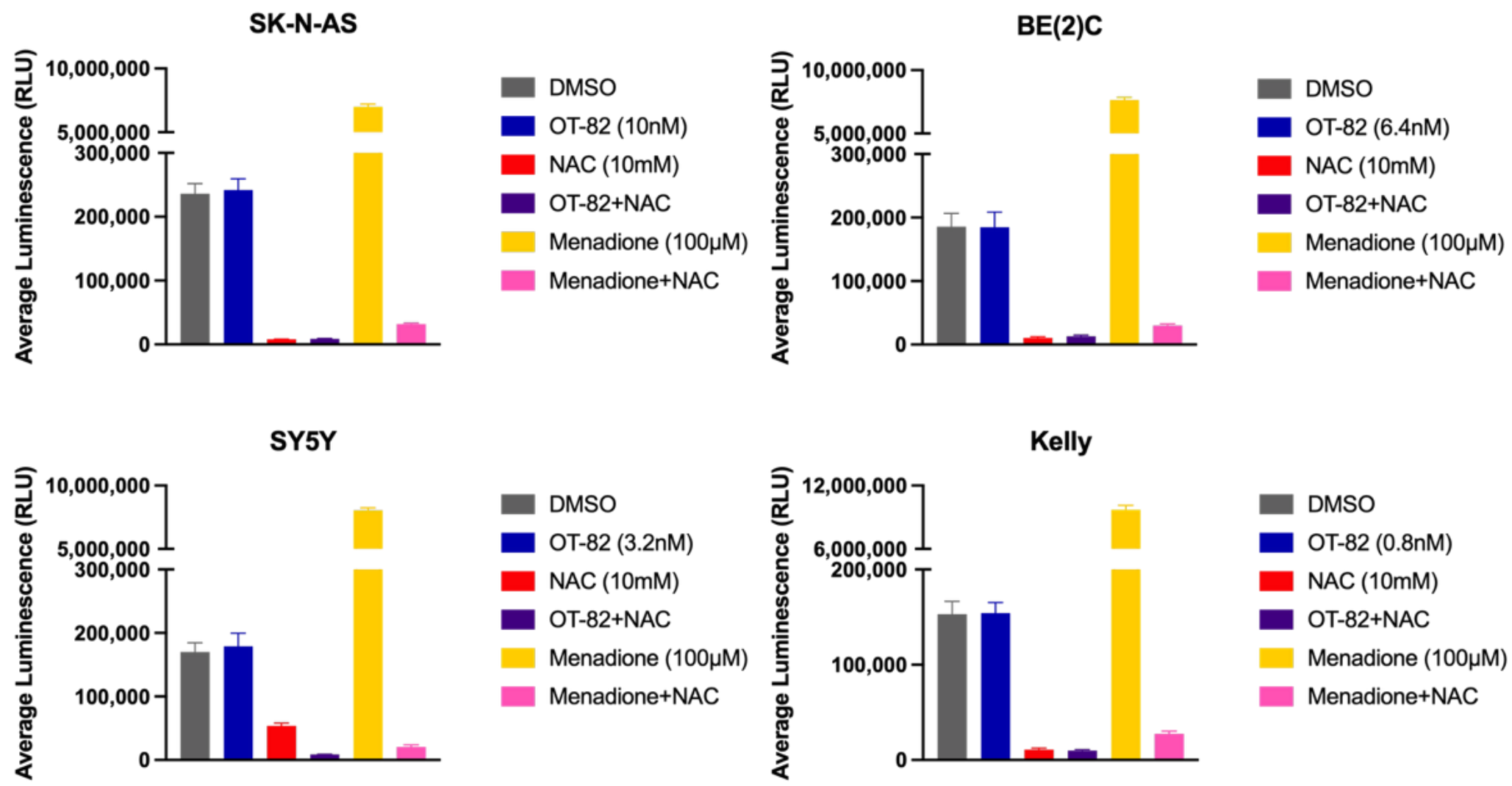

Supplemental Figure S6. (**A**) Cell cycle analysis by flow cytometry of NB cells treated with DMSO or OT-82 (SK-N-AS 10nM, BE(2)C 6.4nM, SY5Y 3.2nM, and Kelly 0.8nM) at 24h, 48h, and 72h showing proportion of cells in sub G1 phase (green), G1 phase (pink), S phase (purple), and G2/M phase (blue). Each experiment was performed at least 3 times with 2 technical replicates. (**B**) Representative IncuCyte images of NB cells at 24h and 96h treated with DMSO or OT-82 (SK-N-AS 10nM, BE(2)C 6.4nM, SY5Y 3.2nM, Kelly 0.8nM). (**C**) GSEA analysis of apoptosis pathway comparing OT-82 treated NB cells (SK-N-AS 10nM, BE(2)C 6.4nM, SY5Y 3.2nM, and Kelly 0.8nM) against control at 24h. (**D**) Flow plots of 4 NB cell lines after 24h, 48h, and 72h of DMSO or OT-82 treatment (SK-N-AS 10nM, BE(2)C 6.4nM, SY5Y 3.2nM, and Kelly 0.8nM). X axis represents percent of cells stained with annexin; Y-axis represents percent of cells stained with propidium iodide. Each experiment was performed at least 3 times with at least 2 technical replicates. (**E**) Immunoblot analysis of phosphorylated RIP1, RIP1, phosphorylated MLKL, and MLKL protein levels in NB cells treated for 48h with DMSO or OT-82 (BE(2)C 6.4nM, SK-N-AS 10nM, SY5Y 3.2nM, and Kelly 0.8nM). Each experiment was performed at least 3 times. (**F**) ROS-Glo analysis of NB cells treated with DMSO or OT-82 (SK-N-AS 10nM, BE(2)C 6.4nM, SY5Y 3.2nM, and Kelly 0.8nM) for 24h. Each experiment was performed at least 2 times with 3 technical replicates.
