# Supplementary material for: Therapeutic NAMPT inhibition reveals a targetable metabolic vulnerability in neuroblastoma
Source: J Exp Clin Cancer Res. 2026 Aug 5;45:227. doi: 10.1186/s13046-026-03793-5 (PMC13726087; doi:10.1186/s13046-026-03793-5)
Supplement: Supplementary file 9 [file 13046_2026_3793_MOESM7_ESM.pdf]

Supplemental Figure S7

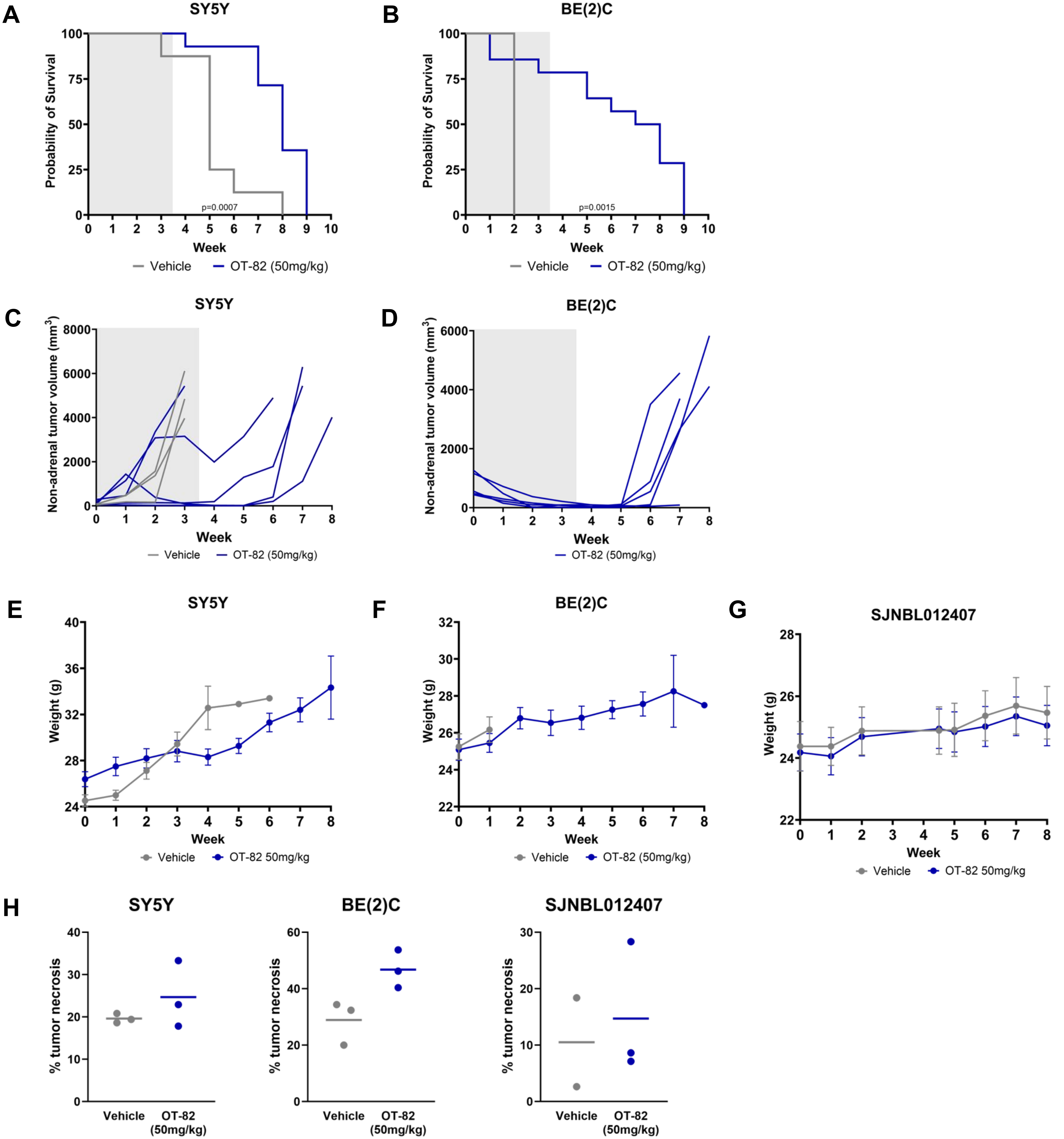

Supplemental Figure S7. (**A**) Kaplan-Meier curve of SY5Y-bearing xenografts in the vehicle (gray) or OT-82 treatment group (blue). Log-rank (Mantel-Cox) test p=0.0007. (**B**) Kaplan-Meier curve of BE(2)C-bearing xenografts in the vehicle (gray) or OT-82 treatment group (blue). Log-rank (Mantel-Cox) test p=0.0015. (**C**) Tumor volume of the non-adrenal tumor by ultrasound imaging in SY5Y-bearing xenografts in the vehicle (gray) or OT-82 treatment group (blue). (**D**) Tumor volume of the non-adrenal tumor by ultrasound imaging in BE(2)C-bearing xenografts in the vehicle (gray) or OT-82 treatment group (blue). Average mouse weights of (**E**) SY5Y-bearing xenograft models, (**F**) BE(2)C-bearing xenograft models, and (**G**) SJNBL012407-bearing xenograft models. Error bars represent SD. (**H**) Percent tumor necrosis in H&E stained tissue collected 2 hours after the third dose of vehicle (gray) or OT-82 (blue).
