# Supplementary material for: Therapeutic NAMPT inhibition reveals a targetable metabolic vulnerability in neuroblastoma
Source: J Exp Clin Cancer Res. 2026 Aug 5;45:227. doi: 10.1186/s13046-026-03793-5 (PMC13726087; doi:10.1186/s13046-026-03793-5)
Supplement: Supplementary file 10 [file 13046_2026_3793_MOESM8_ESM.pdf]

Supplemental Table S1: Key Reagents

REAGENT	IDENTIFIER/OTHER INFORMATION	SOURCE
**Cell lines**		
SK-N-AS	CRL-2137<br>RRID: CVCL_1700	American Type Culture Collection (ATCC)
BE(2)C	CRL-2268<br>RRID: CVCL_0529	ATCC
Kelly	RRID: CVCL_2092	NCI POB
SY5Y	RRID: CVCL_0019	NCI POB
IMR-32	RRID: CVCL_0346	NCI POB
IMR-5	RRID: CVCL_1306	NCI POB
KCNR	RRID: CVCL_7134	NCI POB
LAN5	RRID: CVCL_0389	NCI POB
**Patient-derived xenograft cell lines**		
SJNBL012407		Described in [1].
SJNBL013762		Described in [1].
**Tissue culture**		
RPMI 1640 Medium	11875085	Thermo Fisher Scientific
Fetal Bovine Serum, heat-inactivated	12306C-500ML	Sigma Aldrich
Penicillin-Streptomycin	15140-122	Thermo Fisher Scientific
L-glutamine (200mM)	25030081	Thermo Fisher Scientific
Dulbecco's Phosphate Buffered Saline	14190-144	Thermo Fisher Scientific
Trypsin-EDTA (0.25%)	252000-56	Thermo Fisher Scientific
Trypsin-EDTA (0.05%)	25300-054	Thermo Fisher Scientific
**Compounds**		
OT-82	NAMPT inhibitor	OncoTartis
Daporinad	S2799	SelleckChem
KPT-9274	S84444	SelleckChem
B-Nicotinamide Mononucleotide (NMN)	16411	Cayman Chemical
Oligomycin	495455	Sigma Aldrich
Trichostatin A	T8552	Sigma Aldrich
Propidium Iodide	P4864-10ML	Sigma Aldrich
DNAse-free RNAse A	R5503	Sigma
N-acetyl-L-cysteine	A9165	Sigma Aldrich
Menadione	M5625	Sigma Aldrich

Puromycin	P8833-25mg	Sigma Aldrich
**Cell Lysis**		
Halt Protease and Phosphatase Inhibitor Cocktail	78446	Thermo Fisher Scientific
1X RIPA Lysis and Extraction Buffer	89901	Thermo Fisher Scientific
IGEPAL CA-630	I3021-100ML<br>Component of NP40 lysis solution	Sigma Aldrich
1M Tris-HCl (pH 7.5)	46-030-CM<br>Component of NP40 lysis solution	Cellgro
5M NaCl	46-032-CV<br>Component of NP40 lysis solution	Cellgro
**Immunoblotting**		
4X LDS Sample Loading Buffer	NP0007	Thermo Fisher Scientific
10X Sample Reducing Agent	NP0009	Thermo Fisher Scientific
MOPS SDS Running Buffer (20X)	2815928	Thermo Fisher Scientific
NuPAGE 4-12% Bis-Tris Gel	NP0323BOX	Thermo Fisher Scientific
NuPAGE Transfer Buffer (20X)	2801405	Thermo Fisher Scientific
Tween20	P1379-500ML	Sigma Aldrich
10X Tris Buffered Saline	5460-0037	SeraCare
Omniblock Non-Fat Dry Milk	AB10109-01000	American Bio
SuperSignal West Pico Chemiluminescent substrate	34580	Thermo Fisher Scientific
SuperSignal West Femto Chemiluminescent substrate	34095	Thermo Fisher Scientific
**Antibodies**		
NAMPT	11776-1-AP (1:2000)<br>RRID: AB_2298317	ProteinTech
NAPRT	66159-1-1G (1:500)<br>RRID: AB_2881555	ProteinTech
QPRT	TA501524 (1:500)<br>RRID: AB_11125484	Origene
GAPDH	Sc-365062 (1:1000)<br>RRID: AB_10847862	Santa Cruz
p-T172-AMPKα	2535S (1:1000)<br>RRID: AB_331250	Cell Signaling Technology (CST)
p-Ser792-raptor	2083S (1:1000)<br>RRID:AB_2249475	CST
AMPKα	2532S (1:1000)<br>RRID:AB_330331	CST
Raptor	2280S (1:1000)<br>RRID: AB_10694695	CST
p-T530-SIRT1	Ab156585 (1:1000)<br>RRID: AB_3677705	Abcam
SIRT1	9475 (1:1000)<br>RRID: AB_2617130	CST
Poly/Mono ADP Ribose (PAR)	83732 (1:1000)<br>RRID: AB_2749858	CST
Phospho-RIP1	65746 (1:1000)<br>RRID: AB_2799693	CST

RIP1	3493 (1:1000)<br>RRID: AB_2305314	CST
Phospho-MLKL	91689 (1:1000)<br>RRID: AB_2732034	CST
MLKL	14993 (1:1000)<br>RRID: AB_2721822	CST
LC3B	3868 (1:1000)<br>RRID: AB_2137707	CST
Anti-mouse (secondary)	31430<br>RRID: AB_228307	Thermo Fisher Scientific
Anti-rabbit (secondary)	31460<br>RRID: AB_228341	Thermo Fisher Scientific
Anti-mouse (secondary)<br>(necroptosis blots only)	7076S<br>RRID: AB_330924	CST
Anti-rabbit (secondary)<br>(necroptosis blots only)	7074S<br>RRID: AB_2099233	CST
**Extracellular Flux Assays**		
Seahorse XF base media	103334-100	Agilent
Seahorse XF Calibrant Solution, 500mL	100840-000	Agilent
Seahorse 1.0M Glucose Solution	103577-100	Agilent
Seahorse 100mM Pyruvate Solution	103578-100	Agilent
Seahorse 200mM Glutamine Solution	103579-100	Agilent
**Commercial Assays**		
BCA Protein Assay Kit	23225	Thermo Fisher Scientific
NAD/NADH-Glo Assay	G9072	Promega
AllPrep DNA/RNA Micro Kit	80204	Qiagen
RNase-Free DNase Set	79254	Qiagen
Cell Titer-Glo 2.0 Cell Viability Assay	G9241	Promega
SIRT1 Activity Assay	Ab156065	Abcam
CometAssay Single Cell Gel Electrophoresis Assay	4250-050-K	Bio-Techne
Annexin V FITC Assay Kit	600300	Cayman Chemical
ROS-Glo H_2O_2	G8820	Promega
**In Vivo**		
(2)-Hydroxypropyl-B-cyclodextrin (HPBCD)	H107-5G	Sigma

1. Nguyen, R., et al., *Combining selinexor with alisertib to target the p53 pathway in neuroblastoma.* Neoplasia, 2022. **26**: p. 100776.
