# Supplementary material for: Therapeutic NAMPT inhibition reveals a targetable metabolic vulnerability in neuroblastoma
Source: J Exp Clin Cancer Res. 2026 Aug 5;45:227. doi: 10.1186/s13046-026-03793-5 (PMC13726087; doi:10.1186/s13046-026-03793-5)
Supplement: Supplementary file 11 [file 13046_2026_3793_MOESM9_ESM.pdf]

Supplemental Table S2: Molecular features of neuroblastoma models

Cell Line	Model Type	NAPRT Protein Expression	MCYN	ALK	TP53	Other
**SK-N-AS**	Cell line	Present	Non-amplified [1]	WT [2, 3]	Mutant [4, 5]	NRAS mutant [6]
**BE(2)C**	Cell line	Present	Amplified [7]	WT [3]	Mutant [5, 8]	
**SY5Y**	Cell line	Not detected	Non-amplified [9]	F1174L [3]	WT [5, 8]	
**Kelly**	Cell line	Not detected	Amplified [10]	F1174L [5]	Mutant [5]	
**IMR-32**	Cell line	Not detected	Amplified [1]	WT [3]	WT [5]	
**IMR-5**	Cell line	Not detected	Amplified [11]	WT [2]	WT [5]	
**KCNR**	Cell line	Present	Amplified [1]	F1174L [3]	WT [4]	
**LAN5**	Cell line	Not detected	Amplified [7]	R1275Q [3]	WT [5]	
**SJNBL012407_X1**	PDX	Present	Amplified [12, 13]	WT [13]	WT [12, 13]	
**SJNBL013762_X1**	PDX	Present	Amplified [12, 13]	Mutant [13]	WT [13]	FGFR1 mutant [13]

1. Thiele, C.J., *Neuroblastoma*, in *Human Cell Culture: Cancer Cell Lines Part 1*, J.R.W. Masters and B. Palsson, Editors. 1999, Springer Netherlands: Dordrecht. p. 21-53.
2. George, R.E., et al., *Activating mutations in ALK provide a therapeutic target in neuroblastoma*. Nature, 2008. **455**(7215): p. 975-8.
3. De Brouwer, S., et al., *Meta-analysis of neuroblastomas reveals a skewed ALK mutation spectrum in tumors with MYCN amplification.* Clin Cancer Res, 2010. **16**(17): p. 4353-62.
4. Li, Z., et al., *In vitro and in vivo inhibition of neuroblastoma tumor cell growth by AKT inhibitor perifosine.* J Natl Cancer Inst, 2010. **102**(11): p. 758-70.
5. Harenza, J.L., et al., *Transcriptomic profiling of 39 commonly-used neuroblastoma cell lines.* Sci Data, 2017. **4**: p. 170033.
6. Kiessling, M.K., et al., *Targeting the mTOR Complex by Everolimus in NRAS Mutant Neuroblastoma*. PLoS One, 2016. **11**(1): p. e0147682.
7. Mosse, Y.P., et al., *High-resolution detection and mapping of genomic DNA alterations in neuroblastoma*. Genes Chromosomes Cancer, 2005. **43**(4): p. 390-403.
8. Carr, J., et al., *Increased frequency of aberrations in the p53/MDM2/p14(ARF) pathway in neuroblastoma cell lines established at relapse.* Cancer Res, 2006. **66**(4): p. 2138-45.
9. Baker, D.L., et al., *Analysis of nerve growth factor receptor expression in human neuroblastoma and neuroepithelioma cell lines.* Cancer Res, 1989. **49**(15): p. 4142-6.
10. Peet, A.C., et al., *1H MRS identifies specific metabolite profiles associated with MYCN-amplified and non-amplified tumour subtypes of neuroblastoma cell lines.* NMR Biomed, 2007. **20**(7): p. 692-700.
11. Fischer, M. and F. Berthold, *Characterization of the gene expression profile of neuroblastoma cell line IMR-5 using serial analysis of gene expression.* Cancer Lett, 2003. **190**(1): p. 79-87.
12. Nguyen, R., et al., *Combining selinexor with alisertib to target the p53 pathway in neuroblastoma*. Neoplasia, 2022. **26**: p. 100776.
13. Stewart, E., et al., *Orthotopic patient-derived xenografts of paediatric solid tumours*. Nature, 2017. **549**(7670): p. 96-100.
